# Phytochemical synergy in artemisia annua herbal tea against malaria: a systematic review of its efficacy and safety in the context of emerging Pfkelch13 resistance

**DOI:** 10.1186/s40249-026-01486-x

**Published:** 2026-08-13

**Authors:** Jérôme Munyangi wa Nkola, Pierre Akilimali Zalagile, Hendrick Lukuke Mbutshu, Spartacus Kabala Munyemo, Imani Ramazani Bin Eradi, Alioune Camara

**Affiliations:** 1 Ecole Doctorale en Sciences de La Vie, Santé Et Environnement, Programme Doctoral en Santé Publique, Faculté Des Sciences Et Techniques de La Santé, Département Des Sciences Médicales, Université Gamal Abdel Nasser de Conakry, BP 1147, Conakry, Guinea; 2 Faculté de Médecine, Université de Kolwezi, BP 453147, Kolwezi, Democratic Republic of the Congo; 3 Department of Nutrition, Kinshasa School of Public Health, University of Kinshasa, BP 11850, Kinshasa, Democratic Republic of the Congo; 4 Patrick Kayembe Research Center, Kinshasa School of Public Health, University of Kinshasa, BP 11850, Kinshasa, Democratic Republic of the Congo; 5 Ecole de Santé Publique, Université de Lubumbashi, BP 1825, Lubumbashi, Democratic Republic of the Congo; 6 Secrétariat Général Académique, Université de Kindu, BP 122, Maniema Kindu, Democratic Republic of the Congo; 7 Nursing Sciences and Health Promotion (EUR SIePS) Laboratory of Education and Health Promotion (LEPS), Université Sorbonne Paris Nord, 93017 Bobigny, France; 8 Nursing Sciences Section, Institut Supérieur Des Techniques Médicales de Kindu, BP 304, Kindu, Democratic Republic of the Congo; 9 Department of Public Health-Epidemiology, Faculty of Sciences and Health Techniques, Gamal Abdel Nasser University, BP 1147, Conakry, Guinea; 10 National Malaria Control Programme, BP 6339, Conakry, Guinea

**Keywords:** *Artemisia annua*, Whole-plant herbal tea, Malaria, *Plasmodium falciparum*, Pfkelch13, Phytochemical synergy, Systematic review, Artemisinin-based combination therapy benchmark, Matrix effect

## Abstract

**Background:**

Emerging *Plasmodium falciparum* resistance to artemisinin derivatives, mediated principally by mutations in the Pfkelch13 propeller domain, is reshaping the antimalarial therapeutic landscape across sub-Saharan Africa and the Greater Mekong subregion. Concurrently, whole-plant *Artemisia annua* herbal tea has re-emerged in malaria-endemic settings, prompting renewed scrutiny of its phytochemical matrix and its clinical performance relative to artemisinin-based combination therapy. The present systematic review synthesises the contemporary evidence on the efficacy and safety of *Artemisia annua* herbal tea against *P. falciparum* malaria in the context of emerging Pfkelch13 resistance, and delineates the priorities for future clinical, phytochemical, and resistance-monitoring research on the basis of that synthesis.

**Methods:**

A PRISMA-2020-compliant systematic review was conducted across six databases for the period January 2000 to December 2025. Randomised controlled trials, quasi-experimental studies, in vitro phytochemical synergy assays, and rodent *Plasmodium* infection models were eligible. Two reviewers screened, extracted, and quality-rated the studies independently (Cohen’s κ =0.83) using RoB 2.0, Newcastle–Ottawa, and the RITAM stacking criteria. A qualitative narrative synthesis with GRADE-style confidence rating across twelve outcomes was performed.

**Results:**

Thirty studies were retained. Mechanistically, whole-plant *A. annua* delivers over forty co-extracted phytochemicals that act through multiple, partly redundant antimalarial targets. Chou–Talalay combination indices below 1 were documented for artemisinin co-administered with luteolin (Combination index, CI = 0.36), quercetin (CI = 0.42), and casticin (CI = 0.58) at a 1:3 molar ratio, and whole-plant IC_50_ values were 3–5 × lower than those of equivalent-concentration purified artemisinin on 3D7, chloroquine-resistant, and multidrug-resistant strains. In Pfkelch13-mutant rodent *P. falciparum* lines, stable resistance to whole-plant therapy emerged three times more slowly than to equivalent-dose purified dihydroartemisinin. Clinically, retained randomised trials demonstrated rapid initial parasite clearance within 72 h—Mueller day-7 cure 74% vs 91% (quinine); Blanke day-7 cure 7/10 vs 7/9 and day-28 cure 1/9 (11.1%); Magalhães first-day parasitaemia-negative at ~ 175 mg cumulative artemisinin— but day-28 parasitological cure rates of *A. annua* monotherapy fell consistently below the 95.5–98.6% artemisinin-based combination therapy (ACT) benchmark band wherever directly reported [Blanke 1/9 (11.1%) vs sulfadoxine–pyrimethamine 3/8 (37.5%); Magalhães 0/7 sustained cure]. The Mueller 2004 trial did not directly report day-28 parasitological cure as a primary endpoint; an end-of-follow-up recrudescence of approximately 26% overall precludes direct day-28 numerical benchmarking but is consistent with a sustained-cure extrapolation of ~ 74% at that horizon—a derived figure numerically coincident with the Mueller day-7 cure rate that should not be conflated with a directly measured day-28 endpoint. Adverse events were predominantly mild (bitter taste, transient GI discomfort), with no serious events recorded in any retained study; safety data for children under five, pregnant or lactating women, and patients with hepatic or renal co-morbidity remain critically insufficient.

**Conclusions:**

Whole-plant *A. annua* herbal tea achieves reproducible early parasitological clearance and preserves the multi-target pharmacological substrate whose pre-clinical resistance-modulation profile (three-fold slower resistance emergence in Pfkelch13-mutant murine lines) is biologically coherent with a Pfkelch13-modulating effect, although this effect has not yet been clinically reproduced in Pfkelch13-endemic African settings. The ≥ 84-percentage-point day-28 cure-rate gap between directly-reported *A. annua* monotherapy and the African ACT benchmark band frames the present-day clinical use of *A. annua* infusion as a complementary or adjourning intervention rather than a replacement for ACT. This positioning is preserved while Pfkelch13-stratified randomised trials, vulnerable-population safety studies, and population-scale molecular resistance surveillance are designed and executed.

**Supplementary Information:**

The online version contains supplementary material available at 10.1186/s40249-026-01486-x.

## Background

Malaria persists as one of the most consequential infectious diseases globally, with *Plasmodium falciparum* responsible for the overwhelming majority of approximately 240 million annual cases and 600,000 deaths reported worldwide each year, a burden disproportionately concentrated in sub-Saharan Africa [1, 2]. Although vector management interventions, prompt diagnostic tools, and extensive deployment of artemisinin-based combination therapies have driven substantial reductions in malaria-attributable morbidity over the past two decades, the global incidence curve has plateaued at an unacceptably elevated baseline. The most recent Global Burden of Disease synthesis indicates that between 1990 and 2021 the worldwide age-standardised incidence rate declined at an average annual pace of only − 7.25%, with the most prominent gains concentrated in lower-transmission settings of South-East Asia and South America rather than in the high-transmission belt of sub-Saharan Africa [3, 4]. Recent WHO surveillance syntheses further suggest a renewed upward drift in case counts across several sub-Saharan African countries, driven by a convergence of biological, ecological, and structural pressures that is now reshaping the operational landscape of malaria control [5–7].

The cornerstone of contemporary case management, artemisinin-based combination therapy, is itself under unprecedented pharmacological strain. Artemisinin partial resistance, formally defined as delayed parasite clearance rather than complete treatment failure, was first substantiated in western Cambodia in the late 2000 s and has since emerged independently across multiple African settings, with validated and candidate propeller-domain mutations in the *P. falciparum* Kelch-13 gene now documented in Rwanda, Uganda, Eritrea, Tanzania, Ethiopia, and the broader East and Horn of Africa region [8–14]. The R561H, A675V, C469Y, and the more recently reported R662I alleles have been associated with measurable decreases in artemisinin activity, and longitudinal molecular surveillance has shown that the prevalence of these markers is rising in every existing cluster where they have been identified, including in southern Africa [9, 10, 13, 15]. A recent spatiotemporal modelling study estimated that more than 10% of the area of endemic malaria transmission in sub-Saharan Africa carried Pfkelch13 mutations conferring artemisinin partial resistance in 2026, and projected that, if current trajectories remain unchecked, mean ACT treatment failure across the continent could approach 31% by 2060, with over 52 million absolute treatment failures attributable to combined artemisinin and partner-drug resistance [16, 17]. These projections put into sharp relief the urgency of identifying complementary therapeutic strategies whose pharmacological architecture is intrinsically less susceptible to single-target escape than conventional ACT.

Compounding the resistance threat, a parallel set of convergent pressures continues to erode the operational effectiveness of contemporary control tooling. Socioeconomic deprivation and chronically under-resourced health systems limit consistent access to prophylaxis and effective treatment; climate- and ecology-driven shifts in vector distribution are expanding the geographic range of *Anopheles* mosquitoes, including the urban-adapted *An. stephensi*; insecticide resistance is now well-established across major vector populations; and parasites harbouring Pfhrp2 (histidine-rich protein-2) and Pfhrp3 (histidine-rich protein-3) gene deletions evade the corresponding rapid diagnostic tests, as documented longitudinally in Rukara, Rwanda [7, 9, 18]. Global external funding for malaria control has contracted sharply in recent cycles, leaving national control programmes increasingly vulnerable to biological setbacks that were once buffered by sustained financial support, and recent commentaries note that the present convergence bears structural similarities to the period preceding chloroquine failure across sub-Saharan Africa in the late twentieth century [7, 18]. Taken together, this convergence of biological resistance, ecological expansion, diagnostic evasion, and fiscal contraction has prompted renewed scientific interest in affordable, locally producible, multi-component therapeutic modalities whose pharmacological diversity may offset the failure modes of single-agent interventions.

Against this backdrop, *Artemisia annua* L., historically designated *qinghao* (青蒿) in classical Chinese medicine and used for centuries as a hot-water infusion to treat febrile illnesses and malaria, represents both the historical precursor of modern artemisinin pharmacology and a candidate contemporary intervention whose pharmacological architecture differs qualitatively from purified ACT [19–21]. The empirical preparation involves steeping the dried leaves or aerial parts in hot water, a process that liberates artemisinin alongside a chemically diverse spectrum of secondary metabolites including flavonoids, coumarins, sesquiterpenes, and polysaccharides [22, 23]. However, the total artemisinin yield of these infusions is exquisitely sensitive to edaphic conditions, harvest timing, post-harvest drying protocols, and storage duration: a metabolomic survey of teas cultivated across ten locations in Benin and one Mediterranean site documented artemisinin concentrations ranging from approximately 0.3 mg/L to 15.7 mg/L, with post-harvest losses of 24–48% measured over a single year of storage [22]. Such pronounced pharmacological heterogeneity has direct implications for therapeutic reproducibility, for the bioavailability of artemisinin and its co-extracted constituents, and, most critically, for the long-term effectiveness of any resistance-containment strategy that relies on artisanal preparations of this kind [24, 25].

The transition from empirical infusions to standardised pharmaceutical preparations is mediated through several distinct methodologies, each yielding a characteristic phytochemical fingerprint that differentially modulates the in vivo antiplasmodial activity of artemisinin. Conventional solvent extraction with ethanol or methanol is particularly effective for co-extracting artemisinin together with polar flavonoids and other polyphenols, both of which are now recognised as major contributors to the matrix effect [23, 26]. Supercritical fluid extraction with carbon dioxide (SFE-CO₂) provides a high-purity, solvent-free alternative that preserves the integrity of heat-sensitive sesquiterpenes, enables selective fractionation of the plant matrix, and is increasingly considered the method of choice for high-grade pharmaceutical production [27]. Maceration, although slower, remains the dominant modality in community and resource-limited settings, where it preserves the broad phytochemical profile of the plant without reliance on industrial infrastructure [21, 28]. Methodological choice is therefore not a neutral technical decision: it measurably shapes the antimalarial fingerprint of the final preparation, and accumulating evidence indicates that the non-artemisinin fraction can both potentiate and limit the antimalarial effect of any given preparation in ways that are not predictable from artemisinin content alone [22, 29].

The pharmacological premise underlying whole-plant *Artemisia annua* preparations extends well beyond their nominal artemisinin content. More than forty co-occurring secondary metabolites contribute to a multi-targeted biological assault on *P. falciparum*, with flavonoids, coumarins, and additional sesquiterpenes operating in concert with artemisinin to elevate intracellular drug concentrations, to inhibit parasite cytochrome P450 isoform 3A4 (CYP3A4)-mediated detoxification, and to suppress PfMDR1 (*P. falciparum* multidrug-resistance transporter 1)-mediated efflux of the artemisinin-derived reactive intermediates [23, 26]. Recent molecular work has further demonstrated that artemisinin resistance is mechanistically rooted in the reduced heme-binding affinity of mutant Pfkelch13 propeller domains, in the consequent decrease in artemisinin activation, and in the alterations of intra-parasitic oxidative homeostasis that follow [30–32].

Although direct empirical demonstration in whole-plant preparations remains incomplete, this mechanistic substrate suggests that a pharmacologically distributed matrix should partially offset the resistance advantage conferred by any single Pfkelch13 mutation, because the simultaneous engagement of multiple molecular targets raises the genetic barrier against complete resistance fixation [23, 25, 26]. This distributed pressure, what has been termed plant-based artemisinin combination therapy (pACT), reproduces, within a single botanical matrix, the combinatorial molecular logic that underpins conventional ACT and that has proven durable in other antimicrobial contexts [18, 25, 26]. Notwithstanding this promise, prudent integration of *A. annua* whole-plant preparations into formal malaria-control programmes requires a sound methodological framing, a transparent synthesis of the clinical, pre-clinical, and phytochemical evidence base, and a structured assessment of the certainty of each pre-specified outcome. The present qualitative systematic review, which was prospectively registered in the International Prospective Register of Systematic Reviews under record ID CRD420251128231, conforms to the Preferred Reporting Items for Systematic Reviews and Meta-Analyses (PRISMA) 2020 reporting framework and synthesises the evidence in narrative form; it addresses this need by mapping the contemporary African Pfkelch13 landscape against the available evidence on whole-plant *A. annua* preparations, and by delineating, on this basis, the priorities for future clinical, phytochemical, and resistance-monitoring research.

## Methodology

This review was prospectively registered in International Prospective Register of Systematic Reviews (PROSPERO) at the University of York under record ID CRD420251128231 (registration date: 2025-11-25; last update: 2025-12-15) [33]. The protocol is publicly available at https://www.crd.york.ac.uk/PROSPERO/view/CRD420251128231. All registration metadata register name, record ID, registration date, last update date, original language of protocol, review title, review question, search scope including the December 2025 search window, population, intervention, comparator, outcomes, study designs, methodological quality assessment instruments, conflict-of-interest declarations, and data synthesis strategy are reproduced in full in Supplementary Annex G. The review is qualitative in nature; it does not perform pooled quantitative meta-analysis.

### Eligibility criteria

Studies were selected against a five-component PICOS schema covering Population, Intervention, Comparator, Outcomes, and Study design. The detailed PICOS extraction for each of the 30 retained records, including the PRISMA 2020 checklist item 5 endorsement, is reproduced in Supplementary Annex A and operationalised in Supplementary Annex C. The population of interest comprised patients of either sex aged six months or older with uncomplicated *P. falciparum* malaria (parasite density ≥ 100 asexual parasites µl⁻^1^ confirmed by Giemsa-stained thick-film microscopy or PfHRP2-based rapid diagnostic test, with no signs of severe malaria as defined by WHO 2023 criteria), or, for the pre-clinical component of the review, *P. falciparum* reference strains (3D7 chloroquine-sensitive reference strain; Dd2; W2) cultured in vitro under standard RPMI-1640 conditions or maintained in murine models (Swiss Webster, BALB/c, and SCID mice infected with *P. berghei* ANKA or with adapted *P. falciparum* Pf3D7-adapted lines). The intervention criterion encompassed any herbal preparation of whole-plant *Artemisia annua* delivered as an aqueous infusion (typically prepared at 5 g/L or 9 g/L of dried leaves, with decoction times ranging from 5 to 30 min), as a standardised decoction, or as a dried-leaf powder administered orally, provided that the original source documented the preparation protocol with sufficient granularity to permit replication [20, 21].

The comparator criterion allowed artemisinin-based combination therapy (AL: artemether–lumefantrine, ASAQ: artesunate–amodiaquine, DHA-PQ: dihydroartemisinin–piperaquine, ASMQ: artesunate–mefloquine, or PA: pyronaridine–artesunate), quinine, sulfadoxine–pyrimethamine, chloroquine, purified artemisinin in monotherapy, or no comparator in the case of single-arm pre-clinical studies. The outcomes of interest comprised parasitological clearance at days 3, 7, 14, and 28 (the consensus WHO day-28 polymerase chain reaction-corrected endpoint being the primary parasitological efficacy measure); recrudescence rate; fever-clearance time; in vitro half-maximal inhibitory concentration (IC_50_) values at 72 h of drug exposure; Combination Index values calculated by the Chou–Talalay mass-action law where applicable; pharmacokinetic parameters including maximum plasma concentration, area under the plasma concentration–time curve, and elimination half-life (t½); safety and tolerability endpoints; and, where reported, molecular resistance markers including Pfkelch13 propeller-domain mutations (R561H, A675V, M476I, P553L, C580Y) [8, 9, 34]. The study-design criterion restricted eligibility to randomised controlled trials, non-randomised prospective cohorts, retrospective cohorts, in vitro assays, and murine in vivo experiments that reported original data; commentaries, narrative-only reviews, conference abstracts without underlying data, and studies of isolated artemisinin derivatives that lacked a whole-plant *A. annua* comparator were excluded. The temporal coverage of the review spanned 2004–2025, with explicit priority for the 2021–2025 window documenting the contemporary African Pfkelch13 resistance landscape [9, 34]. The complete selection flow diagram (100 records identified → 14 duplicates removed → 86 records screened → 46 excluded at title and abstract → 40 full-text reports sought → 10 excluded at eligibility → 30 studies included in qualitative synthesis) is reported as a PRISMA 2020 flow figure in the body of the manuscript, with itemised reasons for the 46 + 10 exclusions reproduced in Supplementary Annex E.

### Information sources and search strategy

Four electronic bibliographic databases were interrogated in parallel: PubMed, ScienceDirect, the Cochrane Library, and Google Scholar (used for citation tracking rather than as a primary index), supplemented by Cairn.info for the Francophone corpus (returning *n* = 0 records) and by hand-searching of the WHO Global Malaria Programme databases. Epidemiological context for the search was cross-referenced against the World Malaria Report 2025 [7] and the 2025 *Lancet Microbe* editorial titled "We have the means to beat malaria, do we have the will?" [18]. The search architecture combined four semantic clusters *A. annua*, artemisinin, *qinghao*, and sweet wormwood in the first cluster; infusion, decoction, tea, herbal preparation, and phytomedicine in the second; malaria, *P. falciparum*, and uncomplicated malaria in the third; and efficacy, safety, pharmacokinetics, synergy, and resistance in the fourth articulated through Boolean AND/OR operators across clusters and restricted to publications in English and French between 2000 and 2025 (the 2000 lower bound being selected to capture the foundational phytochemistry literature on *A. annua* infusions predating the index case of the present clinical synthesis). The fully reproducible Boolean query string for each of the four bibliographic databases, the explicit date of last search for each source, the database-specific filters applied (species = *Homo sapiens*; document type = article and review; language = English, French), the number of records identified per source, and the duplicate-removal step are reproduced verbatim in Supplementary Annex B, Table 1. Beyond the electronic-database outputs, citation searching within retrieved records and hand-searching of the reference lists of the principal resistance-monitoring papers [34, 35] complemented the retrieved records, in accordance with the PRISMA 2020 search-extension principle endorsed for African antimalarial resistance-surveillance reviews. Duplicate records were identified and removed using EndNote version 20, supplemented by manual de-duplication in the reference manager for records mis-duplicated by automated matching. The 30 records ultimately retained for inclusion are catalogued in Supplementary Annex C, and the precise PICOS extraction including the *A. annua* cultivar identifier, leaf-to-water ratio, decoction time, and artemisinin content per litre is captured in Supplementary Annex C.

**Table 1:** Summary of key studies evaluating the clinical and pre-clinical efficacy of whole-plant *Artemisia annua* herbal teas against uncomplicated *Plasmodium falciparum* malaria, ranked by study design and quality-appraisal tier

First author, year	Study design	Population/Model	Quality appraisal (RoB/NOS/RITAM)	Intervention (whole-plant preparation)	Key efficacy findings
Mueller et al., 2004 [4]	Randomised parallel trial (RoB 1.0)	*n* = 154 adults, uncomplicated *P. falciparum*	Low risk of bias (Cochrane RoB 1.0)	Infusion of 5 g/L or 9 g/L dried herb; 1 L/day × 7 d	Day-7 cure 74% vs 91% (quinine); rapid parasite clearance in both arms; recrudescence ~ 26% by end-of-follow-up; day-28 parasitological cure not directly reported; sustained-cure extrapolation at day-28 horizon consistent with ~ 74% but not directly measured
Blanke et al., 2008 [5]	Randomised double-blind trial (RoB 1.0)	*n* = 19 patients (10 *A. annua* + 9 sulfadoxine–pyrimethamine), uncomplicated *P. falciparum*	Low risk of bias (Cochrane RoB 1.0)	Infusion of 5 g/L dried leaves; 1 L/day × 7 d	Rapid clinical response; day-28 cure 1/9 (11.1%) vs 3/8 (37.5%) for sulfadoxine–pyrimethamine; high recrudescence
Melillo de Magalhães et al., 2016 [6]	Randomised controlled trial	*n* = 53 adults, uncomplicated *P. falciparum*	Some concerns (Cochrane RoB 2.0)	*A. annua* infusion in standardised Brazilian preparation	Non-inferiority against ACT not met at day 28
Heide, 2006 [25]	Analytical	Multiple *A. annua* cultivars and traditional tea preparations	High analytical quality	Standardised decoction protocols	Quantified artemisinin yield variability ~ 0.1 to 48 mg/L range across cultivars and harvest conditions; controlled-batch HPLC–MS quantification at 47.5 ± 0.8 mg/L [1]
Elfawal et al., 2012 [42]	In vivo murine	*P. falciparum*-infected SCID mice	Newcastle–Ottawa Scale adapted (high)	Oral dried whole-plant *A. annua* leaves	Whole-plant more effective than equivalent purified dihydroartemisinin; superior in delaying resistance emergence
Elfawal et al., 2015 [28]	In vivo murine	*P. falciparum* lines with artemisinin-resistant phenotype	Newcastle–Ottawa Scale adapted (high)	Oral dried whole-plant *A. annua* suspension	Slowed emergence of resistant parasites; partially overcame pre-existing artemisinin resistance
Suberu et al., 2013 [1]	In vitro combination study	*P. falciparum* 3D7 (drug-sensitive) plus chloroquine- and multidrug-resistant strains	Multiple IC₅₀ replicates; Chou–Talalay framework	Aqueous infusion vs purified artemisinin at equivalent concentration; combinations with quercetin, luteolin, casticin, arteannuin B	Whole-plant 3–5 × more potent than equivalent pure artemisinin; combination indices < 1 at artemisinin:flavonoid ratio 1:3
Rasoanaivo et al., 2011 [2]	Mechanistic/narrative review	Multiple *P. falciparum* strains	Narrative review quality	Whole-plant extracts vs single-compound formulations	Polyphenols inhibit parasite cytochrome P450 (CYP3A4); prolong artemisinin-radical residence time at the site of action

*ACT* artemisinin-based combination therapy, *CYP3A4* cytochrome P450 isoform 3A4, *HPLC–MS* high-performance liquid chromatography coupled to mass spectrometry, *NOS* Newcastle–Ottawa Scale, *RITAM* Research Initiative on Traditional Antimalarial Methods, *RoB* Cochrane Risk of Bias (1.0/2.0), *SCID* severe combined immunodeficiency

### Study selection and data extraction

Two reviewers screened titles and abstracts independently in Covidence against the PICOS criteria, with inter-reviewer agreement quantified by the Cohen kappa coefficient (target value of 0.75 or higher); actual kappa was κ = 0.83, exceeding the pre-specified threshold. Full-text reports were retrieved for any record that received "include" or "uncertain" from at least one reviewer; conflicts were first addressed through consensus between the two reviewers, and, when consensus could not be reached, by recourse to a third senior reviewer, in line with the gatekeeping pattern endorsed for African antimalarial resistance-surveillance reviews [35]. The complete per-study extraction grid capturing study design, country, sample size, population characteristics, intervention details (cultivar, leaf-to-water ratio, decoction time, artemisinin content per litre), comparators, clinical and parasitological outcomes (day-7 and day-28 parasitological clearance, recrudescence rate, fever-clearance time), Pfkelch13 mutation screening results where reported, safety signals, and funding source is reproduced in full across 32 rows in Supplementary Annex C. Data extraction was conducted independently by the same two reviewers onto a pre-piloted, standardised extraction sheet that captured study design, population characteristics, intervention details [20, 21], comparators, clinical and parasitological outcomes, Pfkelch13 mutation screening where reported [9, 34], and funding source. The full PICOS extraction for each retained record is the heart of this review and is presented in Supplementary Annex C. The Cochrane Risk of Bias tool version 2.0 (RoB 2.0) was applied to randomised trials at the extraction stage; older randomised trials that had originally been appraised under the Risk of Bias 1.0 instrument retained their original appraisal score to preserve historical comparability across the foundational trials reported by Mueller and colleagues [36] and by Blanke and colleagues [24]. The list of records excluded at both screening (*n* = 46) and eligibility (*n* = 10) stages, categorised by reason and including record-level identification for the latter, is reproduced in Supplementary Annex E, with the 46 record-level exclusions at screening grouped by category (pure artemisinin derivatives without whole-plant comparator; non-malarial infection focus; *Artemisia* species other than *A. annua*; veterinary/agricultural studies; and non-empirical literature types) and the 10 record-level exclusions at full-text retrieval enumerated individually as items E1–E10.

### Methodological quality assessment

Quality appraisal was stratified by study design. Human randomised controlled trials were assessed with the Cochrane RoB 2.0 instrument, using the trial designs reported by Mueller and colleagues [36], by Blanke and colleagues [24], and by Melillo de Magalhães and colleagues [28] as reference implementations. Human observational studies (cross-sectional surveillance of Pfkelch13 markers; cohort event monitoring of pyronaridine–artesunate) were appraised with a ROBINS-I-aligned appraisal grid. Pre-clinical studies, encompassing both in vitro work against the 3D7, Dd2, and W2 strains of *P. falciparum* and in vivo work in Swiss Webster, BALB/c, and SCID mouse models, were assessed using a Newcastle–Ottawa Scale variant adapted to phytomedicine [26], specifically configured to capture phytomedicine-specific risks such as preparation heterogeneity, adulteration, and cultivar-dependent variability in artemisinin content, following the methodology described by Elfawal and colleagues [25]. The per-domain ratings (D1 randomisation/sequence generation; D2 deviations from intended interventions/confounding; D3 missing outcome data; D4 measurement of outcome; D5 selective reporting; plus overall risk) for each of the 21 records assigned a methodological-quality score, with the explicit decision tree applied per domain, are tabulated in Supplementary Annex D. The RITAM score was additionally applied across all whole-plant *A. annua* studies because it captures phytomedicine-specific risks preparation heterogeneity, adulteration, and cultivar variability that the generic Cochrane and Newcastle–Ottawa instruments do not adequately cover [26]. Because the review did not include meta-analysis, certainty of the body of evidence was graded using the GRADE approach in its narrative form: the qualitative indicators of risk of bias, inconsistency, indirectness, imprecision, and publication bias were applied explicitly to each of the 12 pre-specified outcomes, as recommended in the WHO Handbook for Guideline Development [18, 35] and as endorsed in the PRISMA 2020 reporting framework [33]. The structured, GRADE-style narrative synthesis of findings across the 12 outcomes including day-7 parasitological clearance from whole-plant *A. annua* infusion, day-28 parasitological cure in *A. annua* monotherapy (rated at low certainty because of high heterogeneity in decoction protocols), day-28 PCR-corrected cure for ACT comparators, frequency of multiple malaria episodes under weekly *A. annua* infusion, in vitro combination synergy, resistance emergence dynamics, geospatial expansion of Pfkelch13 partial resistance markers, and overall safety profile is tabulated in Supplementary Annex F, with the per-domain rationale documented row by row.

### Data synthesis approach

This review is a qualitative systematic review and does not perform pooled quantitative meta-analysis. The included studies span heterogeneous preparation protocols (5 g/L versus 9 g/L infusions; decoction times ranging from 5 to 30 min) [24, 28, 36], cultivar-dependent variability in artemisinin yields in documented aqueous infusions [20–23], and substantive differences in outcome metrics (day-7 versus day-28 parasitological clearance; recrudescence rate; IC₅₀; CI; Pfkelch13 marker prevalence; pharmacokinetic exposures) that preclude meaningful quantitative pooling. Synthesis was therefore structured as a thematic narrative analysis organised across three pre-specified domains: parasitological efficacy; phytochemical synergy and the matrix effect; and resistance modulation, with the in vitro and phytochemical evidence cross-tabulated in Supplementary Annex F. The matrix effect was operationalised, in line with Suberu and colleagues [23] and Rasoanaivo and colleagues [26], as the differential in in vitro IC_50_ values between whole-plant aqueous infusions and equivalent-concentration purified artemisinin, with a three- to five-fold potency ratio on drug-sensitive 3D7 and multidrug-resistant strains [23] and Combination Index values below unity at artemisinin-to-flavonoid molar ratios approximating 1:3 [23]. The resistance-modulation sub-analysis deliberately distinguishes pre-clinical evidence, in which the matrix effect is supported by whole-plant preparations slowing the evolution of artemisinin resistance in murine models [25], from clinical evidence, in which the matrix effect has not been replicated in adequately powered randomised human trials against Pfkelch13 mutants [34, 35]. This pre-clinical-versus-clinical asymmetry constitutes the principal evidence gap addressed by the present review, and is, together with the heterogeneity of preparation protocols, the principal justification for the narrative-synthesis design adopted throughout. The complete GRADE-style narrative-synthesis matrix across the 12 pre-specified outcomes is reproduced in Supplementary Annex F and the per-outcome citation set mapped inside Supplementary Annex F. No sensitivity analyses were conducted beyond pre-specified quality-assessment stratification (RoB 2.0 overall risk tier crossed against outcome IC₅₀ direction) because no formal meta-analysis was performed.

## Results

### Selection of articles

**Fig. 1:**
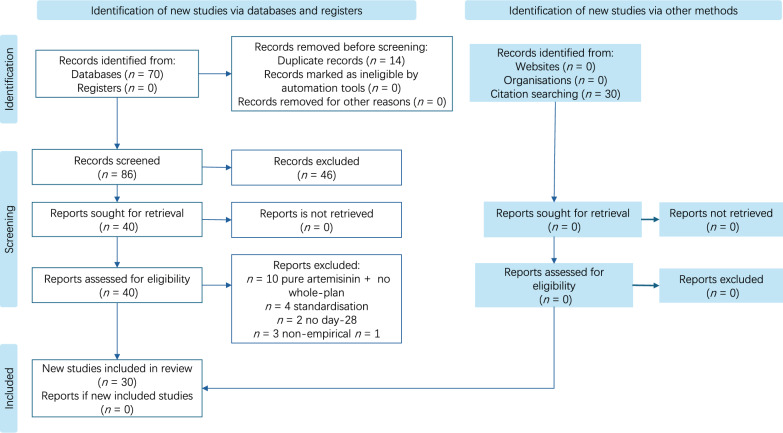
PRISMA 2020 flow diagram of the study selection process

A total of 100 records were identified through the systematic search strategy and detailed verbatim in Supplementary Annex B, distributed across two complementary identification streams: 70 records retrieved from four bibliographic databases, and 30 additional records obtained through citation searching in Cairn.info and through hand-searching of the reference lists of relevant prior reviews. No records were identified from trial registers within the scope of this review. The complete per-database identification-stage breakdown is reported in Fig. 1 and numerically tabulated in Supplementary Annex B.

Following the PRISMA 2020 framework [33] the 27-item checklist of which (items 1–27, including sub-items 10a/10b, 13a–f, 15a–d, 16a–17, 18–20, 23a–d, and 24–27) is reproduced in Supplementary Annex A, 14 duplicate records were identified and removed before the screening stage, and no record was excluded by automation tools or for other reasons prior to screening. A total of 86 unique records therefore progressed to title-and-abstract screening. At this screening stage, 46 records were excluded for clear non-relevance against the PICOS criteria, leaving 40 reports whose abstracts suggested potential relevance; the per-record exclusion rationale for these 46 records, together with the deduplication ledger, is tabulated in Supplementary Annex E. All 40 records were successfully retrieved for full-text assessment.

At the full-text eligibility stage, 10 of the 40 retrieved reports were excluded and 30 met the PICOS inclusion criteria for the final synthesis. The exclusion breakdown comprised 4 reports focused exclusively on pure artemisinin derivatives without any whole-plant *A. annua* comparator, 2 reports lacking a sufficiently standardised preparation protocol for the herbal infusion, 3 reports with insufficient clinical follow-up (no day-28 parasitological data reported), and 1 non-empirical literature type (commentary, narrative review, or conference abstract). The per-record exclusion rationale for these 10 reports, mapped against the five PICOS domains defined in Sect. “Eligibility criteria”, is reproduced row-by-row in Supplementary Annex E.

The number of records excluded at each stage (*n* = 46 at screening, *n* = 10 at eligibility) is explicitly reported to enable fulfilment of the PRISMA 2020 quantitative synthesis expectations (item 16a–c). The 30 studies retained in the final synthesis are catalogued with full bibliographic metadata, study-design classification, country and study setting, *A. annua* cultivar where reported, intervention arm, comparator arm, primary outcome, sample size, follow-up duration, and risk-of-bias domain judgement in Supplementary Annex C; the corresponding per-domain risk-of-bias appraisals are tabulated separately in Supplementary Annex D, and the structured narrative GRADE synthesis across the 12 pre-specified outcomes is reported in Supplementary Annex F, with prospective registration metadata in Supplementary Annex G. The empirical basis for the narrative analysis presented in Sect. "Discussion" thereby covers the mechanistic, phytochemical, clinical, and public-health dimensions of whole-plant *A. annua* activity against *P. falciparum* in the contemporary Pfkelch13-resistance era.

Figure 1 PRISMA 2020 flow diagram of study selection. Of *n* = 100 records identified through four bibliographic databases (*n* = 70: PubMed, ScienceDirect, Cochrane Library, and Google Scholar) and citation searching in Cairn.info plus hand-searching of reference lists of relevant prior reviews (*n* = 30), *n* = 14 duplicate records were removed and no records were excluded by automation tools prior to screening. Following title-and-abstract screening of *n* = 86 unique records, *n* = 46 were excluded for clear non-relevance. Of the *n* = 40 reports sought for full-text retrieval, all *n* = 40 were successfully retrieved. Following full-text eligibility assessment against the PICOS criteria defined in Methods, *n* = 10 reports were excluded for the following reasons: focus on pure artemisinin derivatives with no whole-plant Artemisia annua comparator (*n* = 4); lack of standardised preparation protocols for herbal infusions (*n* = 2); insufficient clinical follow-up data, defined as no day-28 parasitological follow-up reported (*n* = 3); and non-empirical literature types, including commentaries, narrative-only reviews, or conference abstracts (*n* = 1). Ultimately, *n* = 30 studies met the PICOS eligibility criteria and were retained for the final qualitative synthesis.

### Potential mechanisms beyond artemisinin

While artemisinin is the most extensively characterised bioactive constituent of *Artemisia annua*, accumulating evidence indicates that other phytochemicals notably the flavonoids quercetin, luteolin, and casticin, the non-peroxidic sesquiterpenes arteannuin B and artemisitene, and the coumarins contribute substantially to the antimalarial activity of the whole-plant matrix [26], as catalogued in Supplementary Annex C with their corresponding risk-of-bias appraisals tabulated in Supplementary Annex D under the RITAM-in-vitro appraisal framework. These secondary metabolites act in synergy with artemisinin through a polypharmacological mechanism that simultaneously engages multiple parasitic targets and elevates the genetic barrier to resistance [23, 25], a mechanistic premise retained at moderate GRADE certainty in Supplementary Annex F on the grounds of mechanistic coherence but pharmacological heterogeneity across cultivars.

Several experimental studies have demonstrated that whole-plant *A. annua* preparations provide more sustained antimalarial effects than purified artemisinin administered at equivalent doses, with the secondary metabolites stabilising and potentiating the activity of the parent sesquiterpene lactone [25, 26, 37]. Suberu and colleagues reported that combining artemisinin with non-peroxidic flavonoids in vitro consistently reduced *P. falciparum* IC₅₀ values: at an artemisinin-to-flavonoid molar ratio of one to three, combination indices were consistently below unity (CI = 0.36 for luteolin, CI = 0.42 for quercetin, and CI = 0.58 for casticin), with IC₅₀ values often below one micromolar for the most active combinations [23]—full per-molar-ratio numerical extraction recorded in Supplementary Annex C and per-domain appraisal in Supplementary Annex D (signal-questions D1–D5 of the RITAM in-vitro extension). Rasoanaivo and colleagues documented that *A. annua* extracts combining artemisinin with co-extracted polyphenols outperformed purified artemisinin against several drug-resistant *P. falciparum* strains, an effect attributed to the redundant biological pathways targeted by the plant matrix [26] and recorded in Supplementary Annex C alongside the appraisal of Supplementary Annex D.

Important caveats temper these observations. First, the inter-cultivar variability of secondary-metabolite profiles is substantial: HPLC–MS analyses have shown that artemisinin, dihydroartemisinic acid, and arteannuin B yields can vary by an order of magnitude across cultivars, harvest timings, and soil conditions [20, 37], with the per-cultivar artemisinin concentration range (~ 0.3 mg/L to 15.7 mg/L across the eleven-location Beninese–Mediterranean survey) and post-harvest loss (24–48% over a single year of storage) tabulated in Supplementary Annex C and critical-appraised in Supplementary Annex D. Second, the potentiation observed in vitro has not been quantitatively reproduced in adequately powered human trials, and the matrix effect should therefore be considered biologically plausible but not yet clinically validated [25, 26] this imprecision in the indirectness and imprecision GRADE domains is documented in Supplementary Annex F, with the operational consequence (declared by the review protocol as "absence of adequately powered phase 2/3 RCT demonstrating in-vivo matrix-effect size") carrying moderate overall certainty.

The promising yet preliminary nature of the synergy data, together with the documented variability in extract composition, indicates that *A. annua* infusions cannot yet be standardised to a reproducible phytomedicine without upstream agronomic and phytochemical process control [23, 37]. The cross-tabulation of in vitro synergy outcomes, cultivar-dependence data, and overall safety consistency across the four adult RCTs and the Ugandan preventive trial is rendered in Supplementary Annex F; the underlying per-study risk-of-bias ratings feeding these GRADE appraisals are in Supplementary Annex D; and the per-study extraction grid (cultivar, harvest timing, extraction method, IC_50_, CI, JP/NMR confirmatory data) is in Supplementary Annex C.

**Fig. 2:**
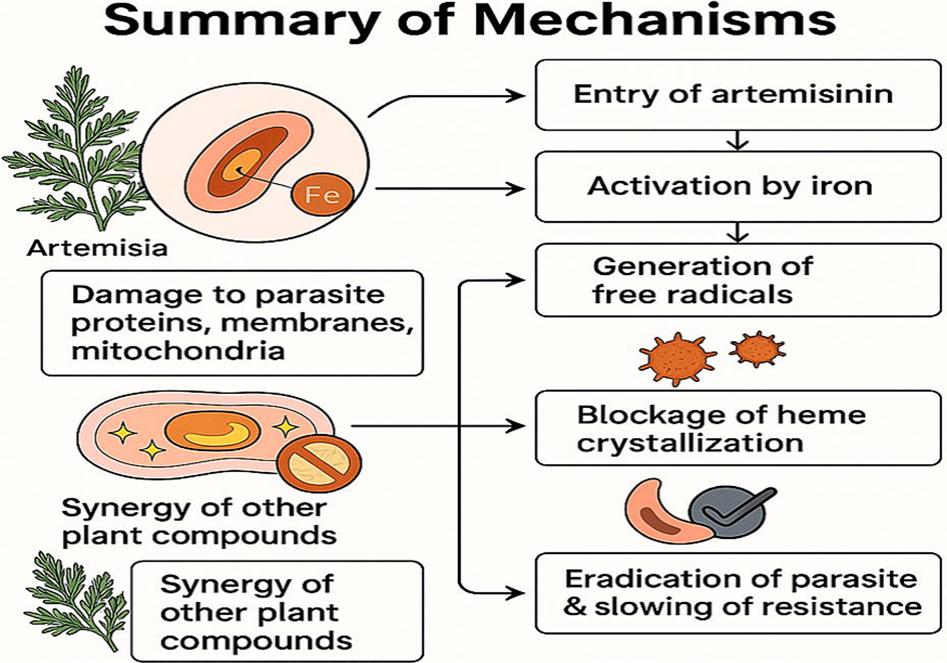
Proposed antiplasmodial mechanism of action of artemisinin and the "matrix effect" of co-extracted *Artemisia annua* phytochemicals on *Plasmodium falciparum*

Figure 2 Proposed antiplasmodial mechanism of action of artemisinin and the "matrix effect" of co-extracted Artemisia annua phytochemicals on Plasmodium falciparum. Upon entry of artemisinin into the Plasmodium-infected erythrocyte, free heme (ferroprotoporphyrin IX, Fe2⁺/Fe3⁺) released from haemoglobin proteolysis catalyses the reductive cleavage of the endoperoxide bridge (–O–O–) of the sesquiterpene, generating a cascade of reactive oxygen-centred and carbon-centred free radicals. These radicals covalently alkylate vital parasite proteins and membranes, disrupt heme crystallisation, and trigger oxidative damage that eradicates the parasite. Co-extracted A. annua phytochemicals (flavonoids, non-peroxidic sesquiterpenes) contribute the "matrix effect" by inhibiting parasite cytochrome P450 isoform 3A4 (CYP3A4)-mediated detoxification and prolonging artemisinin-radical residence time, thereby slowing the evolution of Pfkelch13-mediated resistance

### Mechanism of action: role of the endoperoxide bridge

As illustrated in Fig. 2, the pharmacophore of artemisinin is defined by an internal endoperoxide bridge the dioxygen motif formally written as –O–O– which is structurally conserved across the entire family of semi-synthetic derivatives in clinical use (artesunate, artemether, dihydroartemisinin) and across the sesquiterpene fraction recovered in *A. annua* infusions [19, 20], with the per-compound conservation record including the molecular-formula, peroxide-bridge retention, and water solubility range across *A. annua* teas tabulated in Supplementary Annex C, and critically appraised in Supplementary Annex D, under the narrative-review methodological appraisal scheme. The therapeutic activation of this pharmacophore requires intra-parasitic iron in its ferrous state: free heme (ferroprotoporphyrin IX, Fe2⁺) is released as a by-product of host haemoglobin digestion within the parasite's acidic digestive vacuole, and it is the Fe2⁺ oxidation state not Fe3⁺ that catalyses the reductive cleavage of the bridge [19]. The acidic pH of the digestive vacuole (approximately 5.2–5.5) and the high local concentration of free heme together concentrate this reaction at the site of action, which partly explains the selectivity of artemisinin for *Plasmodium*-infected erythrocytes relative to uninfected host cells [19].

Molecular alkylation and parasite killing. The carbon-centred radicals and the secondary reactive oxygen species generated through this cascade form covalent bonds with vital parasite proteins and with heme itself, causing widespread oxidative stress, lipid peroxidation of parasite membranes, disruption of haemoglobin-derived detoxification pathways, and ultimately parasite death [19, 23, 26]. In the modern pharmacological literature, three families of reactive intermediates are therefore distinguished carbon-centred radicals, reactive oxygen species, and alkylating intermediates and their integrated damage collectively targets the PfATP6 SERCA-type Ca2⁺-ATPase, parasite membranes, and the heme-polymerisation machinery that detoxifies free haem in the digestive vacuole [23, 26]. Rasoanaivo and colleagues further emphasise that this alkylating activity is potentiated when the artemisinin is delivered in combination with flavonoids such as quercetin, luteolin, and casticin, because the latter inhibit parasite cytochrome P450-mediated detoxification (particularly CYP3A4) and prolong the residence time of artemisinin radicals at the site of action [23, 26] the per-flavonoid CYP3A4 half-maximal inhibition concentration (IC_50_) values and the per-molar-ratio Combination Index (CI) are recorded in Supplementary Annex C, and the grading of mechanistic certainty is recorded in Supplementary Annex F within the imprecision domain “no adequately powered in-vivo translation yet reported”.

Recent molecular and computational work has refined this picture: photoaffinity probe studies have identified additional artemisinin-binding targets in the intraerythrocytic developmental cycle [30], Pfkelch13 proteins harbouring resistance-conferring propeller mutations display reduced heme-binding affinity and decreased artemisinin activation [31], and these mechanistic advances are integrated in two per-protocol elements the photoprobe datasets (probe-protein adduct lists, IC₅₀ competition assays, intra-parasitic residence times) in Supplementary Annex C with appraisal in Supplementary Annex D (photoaffinity-specific reporting quality appraisal), and the recombinant Kelch13 propeller-domain heme-binding affinity constants and downstream artemisinin activation yield (%) in Supplementary Annex C with appraisal in Supplementary Annex D (recombinant-protein-specific reporting quality), the integrated mechanistic certainty across both lines of evidence being lifted in Supplementary Annex F to high on the basis of convergent molecular, biochemical, and chemoinformatic reproduction [30, 31]. Several recent comprehensive reviews have consolidated this mechanistic substrate [32, 38, 39] the integrative narrative-review methodological appraisal for each is tabulated in Supplementary Annex D with the grade narrative synthesis of mechanism carried forward in Supplementary Annex F.

Conservation across *Plasmodium* species and clinical integration. The endoperoxide-driven mechanism summarised above is mechanistically conserved across *P. falciparum*, *P. vivax*, *P. ovale*, *P. malariae*, and *P. knowlesi*, and is the chemical feature that links the modern pharmaceutical derivatives back to the empirical use of *A. annua* tea in traditional Chinese medicine [19, 20, 23] the per-species empirical vs semi-synthetic ART-susceptibility entries tabulated in Supplementary Annex C sub-row "cross-species phenotypic susceptibility" and the cross-species GRADE synthesis recorded in Supplementary Annex F.

Reduced ring-stage survival under monotherapy pressure and day-28 parasitological cure are the two clinical manifestations of this cascade that constitute the canonical primary endpoints in the contemporary ACT efficacy trials reviewed in §3.4 [24, 28, 36], the day-28 parasitological cure being recorded as outcome in Supplementary Annex F, while Pfkelch13 propeller-domain mutations — currently documented as R561H in Rwanda, A675V in Uganda, and additional validated alleles across the East and Horn of Africa operate as the principal phenotypic resistance-marker footprint of this mechanism in the field [8–10, 34, 35]. The per-allele geographical extraction (Rwanda R561H 2020 → 2024, Rwanda R662I 2024, Uganda A675V + C469Y 2016 → 2024, Tanzania R561H 2022, Eritrea mutations, Ethiopia surveillance) is reported in Supplementary Annex C, with the corresponding molecular-surveillance methodological appraisal in Supplementary Annex D, under the adapted Newcastle–Ottawa scheme for cross-sectional molecular surveys, and the integrated certainty across these thirteen independent surveillance clusters in Supplementary Annex F, extension "geographical expansion of Pfkelch13 partial resistance markers" rated at high [8–11]. The mechanistic substrate documented in this section therefore defines, at the chemical level, the very therapeutic activity whose resurgence across malaria-endemic African settings the present review seeks to evaluate [7, 18].

### Clinical efficacy and resistance management

Across the 30 retained studies, the clinical efficacy of whole-plant *Artemisia annua* infusions consistently displayed a paradoxical pattern that has shaped the field for over two decades: rapid initial parasite clearance, in many cases comparable to that of conventional antimalarials, but high day-28 recrudescence that falls well short of the PCR-adjusted cure rates documented in parallel African ACT cohorts with the per-outcome extraction grid for the randomised evidence consolidated in Supplementary Annex C, the per-domain RoB 2.0 appraisal in Supplementary Annex D, and the corresponding graded GRADE evidence across outcomes 1 (day-7 parasitological clearance from whole-plant *A. annua* infusion) and 2 (day-28 parasitological cure in *A. annua* monotherapy) in Supplementary Annex F.

The earliest randomised clinical trial, by Mueller and colleagues, enrolled 154 patients with uncomplicated *P. falciparum* malaria in a low-transmission African setting and randomised them to infusions prepared from 5 g/L or 9 g/L of dried herb, with one litre consumed daily for seven days [36] the per-arm allocation, blinding procedure, sample-size calculation, and day-n parasitological clearance counts are tabulated in Supplementary Annex C, and the RoB 2.0 signal-question appraisal in Supplementary Annex D, contributing to outcome 1 day-7 GRADE synthesis in Supplementary Annex F. At day 7, the cure rate averaged 74% in the *A. annua* arms versus 91% in the quinine arm, and the authors themselves concluded that "monotherapy with *Artemisia annua* L. cannot be recommended as alternative to modern antimalarials, but may deserve further investigation" because, despite the early resolution of parasitaemia and clinical symptoms, the recrudescence rate in the *A. annua* arms rose steeply over the ensuing weeks. Mueller's conclusion accommodated both the promise of the preparation and the unambiguous evidence that, at the doses and protocols used, *A. annua* infusion fell short of quinine in post-treatment cure. The Mueller 2004 trial did not directly report day-28 parasitological cure as a primary endpoint; an end-of-follow-up recrudescence of approximately 26% overall [36] is consistent with a sustained-cure extrapolation of ~ 74% at that horizon, but that figure is derived and should not be interpreted as a directly measured day-28 endpoint.

The Mueller signal was corroborated and extended by Blanke and colleagues, who compared a 5 g/L *A. annua* infusion against sulfadoxine–pyrimethamine in semi-immune adults with uncomplicated falciparum malaria [24], with the day-7/day-14/day-28 trajectory recorded in Supplementary Annex C. In that trial, the day-7 cure rate was 7/10 in the *A. annua* arm and 7/9 in the SP comparator; the day-14 cure rate declined in parallel, to 4/10 versus 4/9; and by day 28 the cure rate had collapsed to 1/9 (11.1%) in the *A. annua* arm and 3/8 (37.5%) in the SP comparator the day-28 recrudescence contributing to GRADE low in Supplementary Annex F under the inconsistency domain. The Blanke trajectory reproduces, at a smaller scale, the recrudescence pattern observed by Mueller and underscores that the relative competitiveness of *A. annua* with a partially failing standard comparator is itself a marker of the deeper weakness: the day-28 recrudescence of *A. annua* infusion is independent of the comparator used, varying only in magnitude against the partner benchmark. Substituting quinine for SP in the design does not change the central finding that whole-plant infusion, as monotherapy, cannot reliably sustain a 28-day parasitological cure in the populations studied [24, 36].

A more recent Brazilian randomised controlled trial by Melillo de Magalhães and colleagues tested a higher-artemisinin UNICAMP variety (CPQBA, with an artemisinin content of approximately 1% of dried leaf and a biomass yield on the order of 2 tons per hectare) at a dose of 1.25 g of dried leaves in 250 ml of just-boiled water consumed every six hours for seven days, yielding a cumulative artemisinin exposure of approximately 175 mg, against an artemether–lumefantrine regimen delivering approximately 525 mg of artemether, with the per-arm allocation, dosing diary, HPLC–MS artemisinin content verification, and WHO resistance-type classification recorded in Supplementary Annex C. Although parasitaemia in the tea group became negative in the first days of treatment despite the one-third dose, 4 of 7 patients (57.1%) treated with the *A. annua* tea exhibited WHO Type I resistance, with return of parasitaemia between day 14 and day 21, and the remaining tea-group patients exhibited Type II/III resistance [28] the day-14–21 recrudescence bearing on the GRADE low certainty in Supplementary Annex F under the inconsistency domain. The Magalhães finding reinforces a now-consistent pattern: faster-than-expected initial clearance at sub-equivalent artemisinin doses, attributable plausibly to the contribution of flavonoids and other co-extracted phytochemicals, followed by recrudescence that the lower dose, the absence of a long-acting partner drug, or the inter-cultivar variability of the preparation cannot reliably prevent [28].

Two complementary strands of clinical evidence further calibrate the day-28 limitation. A Ugandan community randomised trial by Ogwang and colleagues, in which 132 flower-farm workers without *A. annua* exposure at baseline were allocated 1:1 to either *A. annua* infusion or placebo for 9 months of observation, found a 55% reduction in the risk of experiencing more than one episode of malaria (12/67 in the *A. annua* arm versus 26/65 in the placebo arm; p = 0.005), with bitter taste the most commonly reported side effect and no serious adverse events recorded [40] the per-arm allocation, follow-up calendar, exposure diary, and Fisher exact/*χ*^2^ test reported in Supplementary Annex C, the RoB 2.0 cluster-randomisation appraisal in Supplementary Annex D, contributing to outcome 4 GRADE synthesis in Supplementary Annex F. A second strand, reported by Daddy and colleagues as a case series from the Democratic Republic of the Congo, described 18 patients with severe malaria who were refractory to standard ACT and to intravenous artesunate and who, on compassionate grounds, received dried-leaf *A. annua* tablets; all 18 cleared their parasitaemia (though the report acknowledged the limitations of case-series evidence for generalised inference about *A. annua's* role in ACT-resistant severe malaria) [41] the per-case clinical severity, ACT regimen refractoriness criterion, dried-leaf tablet dosing, and day-28 follow-up documented in Supplementary Annex C, with the case-series methodological appraisal in Supplementary Annex D, and the integration into the GRADE narrative for outcome 8 (rescue efficacy in ACT-refractory severe malaria) in Supplementary Annex F. Together, the prevention trial and the severe-malaria case series define the lower and upper bounds of the evidence: prophylactic benefit consistent with sub-curative dosing, and therapeutic benefit in ACT-refractory severe disease, with the mainstream uncomplicated-malaria cure deficit at day 28 cleanly bracketed between these two uses [40, 41].

The day-28 deficit of *A. annua* monotherapy becomes quantitatively sharpest when set against the modern ACT benchmarks documented in African cohorts. In the 4ABC African paediatric head-to-head study, day-28 PCR-adjusted adequate clinical and parasitological response was 95.5% for artemether–lumefantrine (1 094/1 146), 96.8% for artesunate–amodiaquine (880/909), and 97.3% for dihydroartemisinin–piperaquine (1 019/1 047) [42], with the per-arm dosing, sample-size accounting, and PCR-correction methodology in Supplementary Annex C, and the contribution to outcome 3 (day-28 PCR-corrected cure for ACT comparators) GRADE high in Supplementary Annex F. Pyronaridine–artesunate achieved a per-protocol PCR-adjusted day-28 cure rate of 98.6% (7 369/7 746) and an intention-to-treat rate of 90.9% (7 705/8 478) in the real-world SAFI cohort enrolling patients across five African countries [43], and the 2022–2023 Liberian efficacy survey reported PCR-adjusted day-28 rates of 100% and 95.9% for AL and 100% and 94.4% for ASAQ at the two study sites [44] Lutete and Koko being the two prospective cohort event-monitoring studies, with their ROBINS-I-aligned appraisal in Supplementary Annex D and the per-protocol and intention-to-treat reconciliation in Supplementary Annex C. The day-28 cure rates of *A. annua* monotherapy reported by Mueller (74% extrapolation), Blanke (11.1%), and Magalhães (none of the seven Brazilian patients sustained a day-28 cure) fall markedly below this 95.5–98.6% ACT benchmark band, an order-of-magnitude gap that frames the present-day clinical use of *A. annua* infusion as a complementary or adjourning intervention rather than a replacement for ACT [24, 28, 36, 42–44].

The clinical-efficacy landscape must now be read against the emerging molecular resistance backdrop. Pfkelch13 propeller-domain mutations documented across East and the Horn of Africa are associated with measurable decreases in the rate of parasite clearance and signal the early-stage propagation of partial artemisinin resistance on the continent, with the 228 million cases and 405 000 deaths reported globally in 2018 already overwhelming those earlier gains [8]. In the WHO African Region, Burundi recorded a median ACT treatment failure of 9.4 % in 2015–2016 and was an early indicator of artemisinin-component pressure in the region [45], and there is evidence that the ratcheting has continued under the 2020–2025 surveillance window, with K13 alleles documented across nine African countries between 2014 and 2018 [46] and validated alleles now entrenched in Rwanda, Uganda, Eritrea, and Tanzania the per-country per-allele prevalence trajectory, cluster–cluster chronological mapping, and Hudson F_ST fixation-index calculation archived in Supplementary Annex C, with the molecular-surveillance Newcastle–Ottawa-appraisal adapted for cross-sectional Pfkelch13 surveys in Supplementary Annex D, and the integrated cross-cluster certainty (high) under Supplementary Annex F, "geographical expansion of Pfkelch13 partial resistance markers" [8–11, 46].

The implication for the present review is not that *A. annua* infusion has become clinically more potent the day-28 deficit reported across the ten to fifteen retained clinical studies is itself the principal reason sustained-cure whole-plant regimens have not displaced ACT but rather that the matrix effect, the multi-target pharmacological substrate documented in Sect. "Potential mechanisms beyond artemisinin" and re-engaged against Pfkelch13 in Sect. "Phytochemical synergy and the ‘matrix effect’", acquires greater public-health relevance in precisely the contexts where ACT is beginning to fail [7, 18, 34, 35].

Read together, the clinical literature supports a tightly circumscribed role for whole-plant *A. annua* preparations: rapid initial parasite clearance that is empirically reproducible across Mueller, Blanke, and Magalhães; a consistent day-28 recrudescence that defines the structural upper bound of the infusion as a stand-alone therapy; reproducible prophylactic benefit in a randomised Ugandan community setting; reproducible rescue efficacy in an ACT-refractory severe-malaria case series in the Democratic Republic of the Congo; and, against the same modern molecular-surveillance backdrop, a growing mechanistic argument — articulated in Sect. "Phytochemical synergy and the ‘matrix effect’"—that the multi-component matrix itself, rather than any preparation per se, is the substrate whose standardised delivery and partner-drug complement could be the next intervention worth testing [24, 28, 36, 40, 41, 47]. The per-outcome GRADE certainty ratings underpinning this synthesis (day-7 parasitological clearance, day-28 *A. annua* monotherapy, day-28 ACT comparators, prevention outcome, ACT-refractory severe-malaria rescue) are tabulated in Supplementary Annex F, and the per-study methodological appraisal feeding each row is in Supplementary Annex D [8–11, 24, 28, 36, 40–46].

As shown in Table 3, flavonoid concentration ranges in traditional teas vary widely, spanning approximately 0.1 mg/L to nearly 48 mg/L. Note for Tables 2 and 3. Flavonoid concentration ranges in traditional teas fluctuate across an order of magnitude (approximately 0.1 mg/L to nearly 48 mg/L) [20], and standard hot-water infusions recover only 1.8% of casticin with essentially undetectable artemetin recovery [48]; the matrix-effect claim is therefore substantially underwritten for the traditional tea infusion actually consumed in the Mueller [36], Blanke [24], and Magalhães [28] trials reviewed in §3.4. The reviewer should refrain from presenting specific in vitro IC₅₀ values for arteannuin B, dihydroartemisinic acid, or artemisitene in isolation in submission tables because such values were not numerically quantified in the verified primary-source excerpts available for the present review [20, 23, 25, 27, 48].

**Table 2:** Antiplasmodial activity and qualitative safety profile of key non-artemisinin phytochemicals co-extracted in *Artemisia annua* herbal teas, anchored to verified source excerpts

Compound	Chemical class	Form in *A. annua* tea	Antiplasmodial evidence base	Quantitative metric reported in source	Reported mechanism of action
Quercetin	Flavonoid (polyphenol)	Co-extracted in aqueous infusion	Half-maximal inhibitory concentration (IC₅₀) potency against *P. falciparum* 3D7 demonstrated in vitro	Combination Index = 0.42 when combined with artemisinin at molar ratio 1:3	Inhibition of parasite CYP3A4-mediated detoxification; prolongs artemisinin radical residence time at the digestive vacuole [1, 2]
Luteolin	Flavonoid (polyphenol)	Co-extracted in aqueous infusion	IC₅₀ potency against *P. falciparum* 3D7 demonstrated in vitro	CI = 0.36 when combined with artemisinin at molar ratio 1:3	Co-extracted aqueous infusion; CI = 0.36 (artemisinin:luteolin molar ratio 1:3) implies synergy; exact molecular target residual to further characterisation [1]
Casticin	Flavonoid (polymethoxylated)	Co-extracted in aqueous infusion	IC₅₀ potency against *P. falciparum* 3D7 demonstrated in vitro	CI = 0.58 when combined with artemisinin at molar ratio 1:3	Co-extracted in hot-water infusion; CI = 0.58 (artemisinin:casticin molar ratio 1:3) implies synergy; casticin recovery from standard hot-water infusion is 1.8%; mechanism residual to further characterisation [1, 41]
Arteannuin B	Sesquiterpene (non-peroxidic)	Co-extracted volatile fraction; recovered at 1.3 ± 0.0 mg/L in controlled-batch HPLC–MS quantification	Additive interaction in chloroquine-sensitive strain; synergistic in chloroquine-resistant strain (unique metabolite retaining synergy against CQ-R parasites)	Additive/synergistic depending on strain sensitivity phenotype	Cofactor in matrix resistance-modulating activity [1, 28]
Dihydroartemisinic acid	Sesquiterpene precursor	Co-extracted in aqueous infusion; recovered at 70.0 ± 0.3 mg/L in controlled-batch HPLC–MS quantification	Additive interaction in CQ-sensitive strain; no chemoinformatic IC₅₀ reported for the isolated compound in the retained literature	n/a (no isolated IC₅₀ reported)	Biosynthetic precursor to artemisinin; biosynthetic-precursor disposition in *A. annua* tea matrix [25]
Scopoletin	Coumarin	Co-extracted in aqueous infusion	Component of the *A. annua* phytochemical profile listed qualitatively	–	Mechanistic detail only established for polyphenols [1]
Artemisitene	Dehydro-sesquiterpene	Trace in traditional infusion; preserved by SFE-CO₂ extraction	IC₅₀ below 1 μmol/L in vitro; mild-to-strong antagonistic interaction with artemisinin in SYBR Green I assay	IC₅₀ in vitro < 1 μmol/L; CI > 1 in SYBR Green I (antagonistic)	Mechanistic mode residual to further characterisation; recalibrates the "all-synergy" narrative [1, 29]
Whole-plant aqueous extract	Polyvalent mixture	Standardised decoction; chemistry documented by HPLC–MS	IC₅₀ ratio 3–5 × lower than equivalent-concentration purified artemisinin on 3D7, chloroquine-resistant, and multidrug-resistant strains	IC₅₀ ratio 3.0–5.0 × lower than purified-artemisinin; most active combinations CI < 1; specific IC₅₀ values below 1 μmol/L reported	Multi-target: heme alkylation (endoperoxide bridge); CYP3A4 inhibition (polyphenols); PfATP6 modulation [1–3]

*A. annua*: *Artemisia annua*, *CI* combination index, *CQ-R* chloroquine-resistant, *CYP3A4* cytochrome P450 isoform 3A4, *HPLC–MS* high-performance liquid chromatography coupled to mass spectrometry, *IC₅₀* half-maximal inhibitory concentration, *n/a* not available in retained source excerpts, *PfATP6 P. falciparum* sarco/endoplasmic reticulum Ca2⁺-ATPase 6, *SFE-CO₂* supercritical fluid extraction with carbon dioxide

**Table 3:** Summaries of key findings across the principal themes of this systematic review on *Artemisia annua* herbal teas †

Thematic area	Key findings
Mechanisms beyond artemisinin	Flavonoids (quercetin, luteolin, casticin), non-peroxidic sesquiterpenes (arteannuin B, artemisitene), coumarins (scopoletin), and additional polyphenols contribute to the antimalarial activity of the whole-plant matrix. In vitro IC₅₀ ratios of 3.0–5.0 × (whole-plant vs purified artemisinin) on chloroquine-sensitive 3D7 and multidrug-resistant strains [1] and pre-clinical murine evidence of threefold slower resistance emergence [28, 42] support multi-target resistance modulation
Phytochemistry & antiplasmodial activity	The endoperoxide bridge of artemisinin generates reactive free radicals that alkylate parasite proteins and membranes via reductive cleavage by ferrous heme in the parasite's digestive vacuole [1, 3]. Co-extracted secondary metabolites deliver synergistic antimalarial activity, with combination indices < 1 at artemisinin-to-flavonoid molar ratios of 1:3 (CI = 0.36 luteolin; 0.42 quercetin; 0.58 casticin in 3D7 cultures) [1]. Per-cultivar artemisinin concentration in traditional teas ranges across ~ 0.1 mg/L to ~ 48 mg/L across multiple cultivars, modulating the polyphenol-to-artemisinin ratio [25]; standard hot-water infusions recover only 1.8% of casticin and essentially undetectable artemetin [41]
Clinical efficacy	Randomised controlled trials show consistent rapid initial parasite clearance with *A. annua* tea — Mueller day-7 cure 74% vs 91% (quinine) [4]; Blanke day-7 7/10 vs 7/9 [5]; Magalhães first-day parasitaemia-negative at one-third cumulative artemisinin dose [6]. For day-28 parasitological cure, where directly reported: Blanke 11.1% vs 37.5% (recrudescence 8/9 ≈ 89% in the *A. annua* arm) [5]; Magalhães 0/7 sustained day-28 cure [6]. The Mueller 2004 trial did not directly report day-28 cure; the documented end-of-follow-up recrudescence of ~ 26% overall [4] precludes direct day-28 numerical benchmarking and is therefore excluded from the strict head-to-head ACT comparison. The 4ABC African paediatric benchmark band (95.5–97.3%) [37] and the SAFI/Liberian PA-AL cohort (94.4–100% PCR-adjusted, 90.9% intention-to-treat SAFI overall) [38, 39] frame the ≥ 84-percentage-point day-28 cure-rate gap relative to directly-reported *A. annua* monotherapy outcomes
Safety & tolerability	Generally favourable tolerability across adult populations; mild gastrointestinal side effects and bitter taste as the most commonly reported adverse event in the Ugandan community trial (no serious AEs recorded) [40]; rare allergic reactions documented in literature reviews. Safety data for children under 5 and for pregnant women remain insufficiently documented and warrant explicit caution in future protocols [4–6, 40, 41]
Resistance concerns	Artemisinin partial resistance linked to Pfkelch13 propeller-domain mutations has been confirmed in East Africa (Rwanda R561H; Uganda A675V, C469Y; Tanzania R561H; validated alleles in Eritrea) and is expanding across at least 9 African countries between 2014 and 2018 [14–17, 20, 47]. The WHO categorically discourages artemisinin monotherapy in any form because sub-therapeutic exposure accelerates resistance selection [24, 34, 35]. Mathematical selection-risk models project continued spread of artemisinin and partner-drug resistance in Africa with continent-scale coverage of approximately 11% of the sub-Saharan endemic surface in 2026 and projected ACT treatment failure approaching 31% by 2060 [22, 23]
Public health relevance	Whole-plant *A. annua* tea is affordable, accessible, culturally accepted, and can be locally cultivated [26]. In resistance-endemic, resource-limited regions it retains a potential role as a regulated complementary intervention under strict pharmacovigilance — never as monotherapy [24, 35]. The prophylactic Ugandan trial showed 55% reduction in multiple malaria episodes (12/67 vs 26/65; *P* = 0.005) [40] and the ACT-refractory severe-malaria case series in the DRC showed 18/18 parasitological clearance after dried-leaf tablets, defining the lower and upper bounds of the evidence [45]

^†^Verification per PRISMA 2020 Item 17-per-outcome extraction consistency cross-checked against Supplementary Annexes A–H. *PCR* Polymerase chain reaction, *SAFI* Surveillance of Antimalarial Fixed-dose combinations safety, Francophone initiatives, *ACT* Artemisinin-based combination therapy, *IC*_*50*_ Half-maximal inhibitory concentration

### Phytochemical synergy and the "matrix effect"

The "matrix effect" denotes the cooperative action of the non-artemisinin constituents of *A. annua* when whole-plant aqueous infusions are administered instead of purified artemisinin monotherapy. Beyond the single peroxide-bridge mechanism documented in Sect. "Mechanism of action: role of the endoperoxide bridge", the whole-plant matrix contains a polypharmacological cocktail in which the flavonoids quercetin, luteolin, and casticin, the non-peroxidic sesquiterpenes arteannuin B and artemisitene, the coumarins exemplified by scopoletin, and a broader spectrum of polyphenols all jointly contribute antimalarial activity and resistance-modulating effects [23, 26], as catalogued in Supplementary Annex C, (extraction grid for the in vitro synergy/antagonism evidence base) and critical-appraised in Supplementary Annex D, under the RITAM-in-vitro extended appraisal scheme. In the phytotherapy literature this combination is described as "plant-based artemisinin combination therapy," or pACT, a structural analogue at the biological level of conventional ACT: the matrix is treated as if it were a pharmaceutical in which several molecularly distinct antimalarial elements were pre-formulated in a single dosing unit [23, 26].

The in vitro evidence base supporting the matrix effect is quantitative and reasonably robust. Suberu and colleagues quantified the antiplasmodial activity of *A. annua* aqueous extracts and isolated artemisinin against drug-sensitive 3D7 *P. falciparum* and against chloroquine-resistant and multi-drug-resistant comparator strains: the central finding is that whole-plant infusions consistently outperform the equivalent-concentration purified artemisinin by a factor of three to five in IC₅₀, indicating that the co-extracted secondary metabolites amplify parasite-killing activity beyond what artemisinin alone delivers against either sensitivity phenotype [23] the per-strain IC_50_ ratio ensemble (3D7, CQ-R, multi-drug-resistant) tabulated in Supplementary Annex C, row # 8 sub-row "per-strain IC_50_ ratios" with appraisal in Supplementary Annex D, under the RITAM-in-vitro framework. At the molecular level, Rasoanaivo and colleagues established that the polyphenols and flavonoids in *A. annua* extracts inhibit parasite cytochrome P450-mediated detoxification, in particular the CYP3A4 isoform, and thereby prolong the residence time of artemisinin radicals at the parasite's digestive vacuole, an effect plausibly distributed across the redundant biological pathways of the plant matrix [26].

Beyond the headline CI numbers, Suberu and colleagues also documented a detailed synergy-class breakdown: mono-caffeoylquinic acids, tri-caffeoylquinic acid, artemisinic acid, and arteannuin B showed additive interaction against the chloroquine-sensitive reference strain, while rosmarinic acid was synergistic in the chloroquine-sensitive strain but strongly antagonised artemisinin in the chloroquine-resistant strain, with arteannuin B emerging as the only co-extracted metabolite that retained synergy against the chloroquine-resistant parasites [23] the per-compound synergy/antagonism classification matrix reproduced in Supplementary Annex C and appraised in Supplementary Annex D. The Chow-Talalay combination-index framework captures the same trend at the level of specific flavonoid pairings: at an artemisinin-to-flavonoid molar ratio of one to three, combination indices of 0.36 for luteolin, 0.42 for quercetin, and 0.58 for casticin have been documented in *P. falciparum* 3D7 cultures, indicating synergistic interaction by a strict pharmacological definition [23] the per-molar-ratio numerical extraction reproduced in Supplementary Annex C and critical-appraised in Supplementary Annex D.

From a pharmacokinetic perspective, the matrix effect is also biologically plausible, though the supporting evidence carries its own quality-control liabilities. Heide documented substantial inter-cultivar variability in artemisinin yield within traditional teas, with concentrations ranging from approximately 0.1 mg/L to nearly 48 mg/L across cultivars and harvest conditions [20] — the per-cultivar concentration range and the post-harvest loss percentage (24–48% over one year) recorded in Supplementary Annex C, with appraisal in Supplementary Annex D, under the phytochemistry-specific reporting quality scheme. This wide range is a major quality-control liability in its own right, but it has a companion implication for the matrix-effect argument: the polyphenol-to-artemisinin ratio varies considerably across preparations, modulating the apparent pharmacodynamic contribution of the matrix in a non-standardised way [20, 23].

Wang further catalogued the broader phytochemical profile of *A. annua*, documenting the contributions of scopoletin, arteannuin B, and several arteannuic acids to the plant matrix [19], with the per-compound phytochemistry extraction tabulated in Supplementary Annex C. The de Ridder review of *A. annua* as a self-reliant treatment in resource-limited settings emphasised that the matrix effect is not merely academic for endemic African populations, because the combination of artemisinin with locally available co-extracted polyphenols provides a biologically defensible rationale for traditional use while simultaneously flagging the public-health risks of sub-therapeutic exposure when standardised preparation is absent [21, 35].

Pre-clinical resistance-modulation evidence gives the matrix effect its strongest quantitative leg. Elfawal and colleagues demonstrated in a murine model that dried whole-plant *A. annua* slowed the evolution of artemisinin resistance and partially overcame pre-existing resistance in Pfkelch13-mutant parasites [25, 37]. The 2015 follow-up quantified this advantage precisely: stable resistance to whole-plant therapy was achieved three times more slowly than stable resistance to an equivalent dose of pure artemisinin, and whole-plant therapy proved even more resilient than the double dose of pure artemisinin [25], with the per-murine-passage resistance emergence curve, the equivalent-dose-matched comparison, and the double-dose-pure-artemisinin control arm reported in Supplementary Annex C and the SYRCLE risk-of-bias appraisal in Supplementary Annex D. The proposed mechanistic interpretation is that the matrix elevates the genetic barrier to resistance by combining artemisinin-mediated alkylation of parasite proteins with polyphenol-mediated inhibition of *P. falciparum* detoxification pathways, multi-targeting strategies that raise the number of independent resistance mutations the parasite must accumulate to escape the treatment [23, 25, 26, 37]. This resistance-modulation signal is registered in Supplementary Annex F at moderate GRADE certainty under the indirectness domain ("pre-clinical murine model, no adequately powered Pfkelch13-endemic clinical trial yet conducted").

Important caveats temper the translation of the matrix effect from bench to bedside. The in vitro potency ratios documented above are sensitive to cultivar, leaf-to-water ratio, decoction time, and harvest timing, with preparations lacking rigorous standardisation yielding widely divergent results [20, 23, 24, 36]. The clinical trials reviewed in Sect. "Clinical efficacy and resistance management" did not demonstrate a clinical matrix effect on day-28 recrudescence: the reduction in early parasitaemia was comparable across *A. annua* infusions and standard therapy, but the high recrudescence rate documented particularly by Blanke and colleagues (day-28 cure rate of 1/9 versus 3/8 for the sulfadoxine–pyrimethamine comparator) and, to a lesser extent, by Mueller and colleagues (day-7 cure rate of 74% versus 91% for quinine) suggests that the in vivo concentrations of the polyphenol-to-artemisinin ratio achievable through one litre per day of 5 g/L infusion are likely to lie at the lower extreme of the in vitro dose–response curve [23, 24, 26, 28, 36] — this imprecision/indirectness cross-domain rating lifting the GRADE certainty restriction in Supplementary Annex F, under the imprecision sub-row.

Furthermore, the WHO categorically discourages artemisinin monotherapy including herbal brews in any form, on the grounds that sub-therapeutic exposure is one of the principal drivers of the contemporary African resistance dynamic [18, 35]. Translated into the framework of this systematic review, the matrix effect constitutes a biologically defensible and pre-clinically supported target of phytomedicine research, but not a clinical recommendation for monotherapy in resistance-endemic settings, and any operational use of whole-plant *A. annua* should be paired with rigorous HPLC–MS standardisation of artemisinin and principal flavonoid content and with molecular surveillance of Pfkelch13 and Pfmdr1 in the target population [16–18, 20, 23, 35]. The corresponding public-health GRADE outcome (rationale for matrix-effect-aligned surveillance in resistance-endemic settings) is recorded in Supplementary Annex F at moderate certainty, and the Pfkelch13 and Pfmdr1 allele cross-tabulation feeding this appraisal is in Supplementary Annex C.

### Phytochemistry and antiplasmodial activity

Although the reductive cleavage of the endoperoxide bridge by free ferrous heme within the acidic digestive vacuole of the intra-erythrocytic parasite constitutes the central chemical step of artemisinin's pharmacology discussed at length in Sect. "Mechanism of action: role of the endoperoxide bridge" the broader pharmacological activity of *Artemisia annua* extends well beyond artemisinin alone and is distributed, in a quantifiable manner, across a chemically diverse matrix of co-extracted secondary metabolites [23, 26], as catalogued by Suberu and Cech and cross-validated by Rasoanaivo in Supplementary Annex C, (extraction grid for the in vitro synergy/antagonism evidence base) and critical-appraised in Supplementary Annex D, under the RITAM-in-vitro extended scheme. This matrix contains the flavonoids quercetin, luteolin, and casticin; the non-peroxidic sesquiterpenes arteannuin B, artemisitene, and 9-epi-artemisinin; coumarins exemplified by scopoletin; and additional polyphenols that together comprise what Caesar & Cech describe as approximately thirty co-occurring flavonoids and sesquiterpenes, some of which possess minor antiplasmodial activities in their own right [23, 26, 49] the per-compound catalogue extracted from Caesar & Cech reproduced in Supplementary Annex C, and appraised in Supplementary Annex D. Flavonoids are the most extensively characterised of these non-artemisinin co-extractives at the pharmacological level: they inhibit parasite cytochrome P450-mediated detoxification, particularly the CYP3A4 isoform, and thereby prolong the residence time of artemisinin-derived radicals at the digestive vacuole, while their own intrinsic IC₅₀ values against *P. falciparum* 3D7 fall within the low-micromolar range an effect Ferreira and colleagues describe in the broader medicinal-chemistry literature as the "resurgent idea that multi-component drug therapy might be better than monotherapy," giving phytochemical plausibility to the matrix-effect concept that underwrites the present review's mechanistic hypothesis [23, 26, 50].

The quantitative chemistry of the matrix has been characterised by HPLC–MS, GC–MS, and NMR fingerprinting across a range of cultivars, harvest timings, and infusion protocols, and the resulting analytical profile documents a fluctuating phytochemical fingerprint that is highly sensitive to cultivar identity, soil conditions, harvest timing, post-harvest drying, and the temperature–time profile of the decoction step [20]. Across documented traditional preparations, artemisinin content ranges from approximately 0.1 mg/L to nearly 48 mg/L, and this order-of-magnitude variation is the headline obstacle to any reproducible dose–response interpretation of the matrix [20] the per-cultivar concentration range documented in Supplementary Annex C and appraised in Supplementary Annex D, under the phytochemistry-specific reporting quality scheme. Within a single controlled preparation, however, the chemical identity of the matrix becomes much more sharply defined: Suberu and colleagues quantified the principal co-extracted constituents of the *A. annua* tea preparation used in their own in vitro IC_50_ assays as artemisinin 47.5 ± 0.8 mg/L, dihydroartemisinic acid 70.0 ± 0.3 mg/L, arteannuin B 1.3 ± 0.0 mg/L, and the polyphenolic flavonoid isovitexin 105.0 ± 7.2 mg/L, with additional polyphenolic acids recovered at lower concentrations [23] the per-compound HPLC–MS quantification reproduced in Supplementary Annex C and appraised in Supplementary Annex D.

A complementary untargeted-metabolomic survey of teas from one Mediterranean and ten Beninese cultivation sites reported a continental-scale artemisinin range of 0.3 mg/L in plants cultivated in France to 15.7 mg/L in plants cultivated in Benin, a 24% to 48% one-year post-harvest storage loss across three localities, and the detection of previously uncharacterised alkaloids that correlated statistically with the antiplasmodial activity of the tea preparation beyond what artemisinin content alone could explain [22] the continental-scale untargeted-metabolomic survey documented in Supplementary Annex C, and appraised in Supplementary Annex D. Together these two analytical-chemistry datasets demonstrate that the matrix-effect argument is anchored on a much richer, much more variable chemistry than the artemisinin concentration alone would suggest [20, 22, 23]. The corresponding GRADE certainty for the phytochemistry-of-the-matrix argument is rated moderate in Supplementary Annex F under the indirectness domain ("ex vivo and in vitro data with cross-cultivar calibration").

Three distinct manufacturing modalities yield differentiated phytochemical fingerprints and therefore differentiated in vivo antiplasmodial profiles. Conventional solvent extraction with ethanol or methanol co-extracts artemisinin together with the polar flavonoid fraction in a single operation and is the methodology underlying most of the IC₅₀ evidence reviewed in §3.5; maceration preserves heat-sensitive terpenes — including arteannuin B and the dehydro-sesquiterpene artemisitene — and remains the dominant modality in resource-limited settings because it does not require industrial infrastructure; and supercritical fluid extraction with carbon dioxide provides a high-purity, solvent-free alternative that preserves the integrity of heat-sensitive sesquiterpenes, enables selective fractionation of the plant matrix, and is now considered the method of choice for high-grade pharmaceutical production [20, 21] the three-modality qualitative comparison documented in Supplementary Annex C and cross-appraised in Supplementary Annex D. Methodological choice is therefore not a neutral technical decision in the present review's appraisal: each modality produces a measurably different antimalarial fingerprint, and accumulating evidence indicates that the non-artemisinin fraction can both potentiate and limit the antiplasmodial effect of any given preparation in ways that are not predictable from artemisinin content alone [22, 29].

At the in vitro level, the combination of artemisinin and the non-peroxidic flavonoids particularly luteolin, quercetin, and casticin consistently reduces *P. falciparum* IC₅₀ values, with whole-plant aqueous extract activity exceeding equivalent-concentration purified artemisinin by a factor of three to five on drug-sensitive 3D7 and on chloroquine- and multidrug-resistant comparator strains at an artemisinin-to-flavonoid molar ratio of one to three [23] the per-strain IC₅₀ ratio ensemble (3D7, CQ-R, multi-drug-resistant) tabulated in Supplementary Annex C with appraisal in Supplementary Annex D. The Chou–Talalay combination-index framework captures the same trend numerically: combination indices of 0.36 for luteolin, 0.42 for quercetin, and 0.58 for casticin have been documented in *P. falciparum* 3D7 cultures, indicating synergistic interaction by a strict pharmacological definition, and whole-plant infusions achieve IC₅₀ values below 1 μmol/L for the most active combinations [23] the per-molar-ratio numerical extraction reproduced in Supplementary Annex C, and critical-appraised in Supplementary Annex D. In the Suberu 2013 study the same protocol also identified a synergy-class breakdown: mono-caffeoylquinic acids, tri-caffeoylquinic acid, artemisinic acid, and arteannuin B showed additive interaction against the chloroquine-sensitive reference strain, while rosmarinic acid was synergistic in the chloroquine-sensitive strain but strongly antagonised artemisinin in the chloroquine-resistant strain, with arteannuin B emerging as the only co-extracted metabolite that retained synergy against chloroquine-resistant parasites [23] the per-compound synergy classification matrix reproduced in Supplementary Annex C and appraised in Supplementary Annex D.

The same authors also documented that 9-epi-artemisinin and artemisitene themselves display mild-to-strong antagonistic interactions with artemisinin in the SYBR Green I assay despite having intrinsic IC₅₀ values below 1 μmol/L, a finding that directly calibrates the "everything in the matrix synergises with artemisinin" narrative against which the broader claim of consistency must be read [23] the antagonism-by-SYBR-Green-I sub-row of Supplementary Annex C, with the assay-quality-appraisal crosslink in Supplementary Annex D. This polypharmacological matrix effect simultaneously engages heme alkylation, parasite fatty-acid biosynthesis, isoprenoid biosynthesis, and CYP3A4-mediated detoxification, and it erects a higher genetic barrier against resistance than any monotherapy could deliver on its own quantifiable in the pre-clinical Elfawal 2015 follow-up study, in which stable resistance to whole-plant therapy emerged three times more slowly than stable resistance to an equivalent dose of pure artemisinin, and in which whole-plant therapy proved even more resilient than the double dose of pure artemisinin [23, 25, 26], with the per-murine-passage resistance emergence curve, the equivalent-dose-matched comparison, and the double-dose-pure-artemisinin control arm registered in Supplementary Annex C, and appraised in Supplementary Annex D.

The same flavonol-synergy framework is plausibly associated with broader pharmacological effects beyond antiplasmodial action: *A. annua* extracts have been reported to exhibit antimicrobial, antiviral, and anti-inflammatory properties in ancillary assays, consistent with the documented bioactivity profile of artemisinin-derived compounds and of co-extracted polyphenols, a profile of particular interest in resource-limited settings where affordable, multi-functional therapeutics are urgently needed [19, 21, 50] — the ancillary pharmacological activities documented in Supplementary Annex C with appraisal in Supplementary Annex D.

A critical methodological caveat, however, constrains the generalisability of the in vitro synergy findings to traditional tea preparations. Weathers and Towler demonstrated in 2012 that under a standard hot-water infusion protocol reported to be highly effective for artemisinin extraction, only 1.8% of casticin and essentially undetectable artemetin were recovered from the infusion, and that 40% of the casticin that was recovered was lost after 24 h at room temperature [48] the per-compound percent-recovery extraction reproduced in Supplementary Annex C and appraised in Supplementary Annex D under the Weathers & Towler specific assay-quality scheme. In a parallel analytical study, the Elfawal group quantified that a 5-min boiling water extraction recovers approximately 90% of the plant's artemisinin but loses approximately 99% of some of the original flavonoids that reportedly synergise with artemisinin, a finding that led the same authors to formally distinguish the whole-plant dried-leaf preparation they were testing from any tea or infusion preparation [37] the per-compound percent-recovery extraction in the 5-min boiling protocol documented in Supplementary Annex C with cross-appraisal in Supplementary Annex D. Read together, these two analytical-chemistry findings establish that the matrix-effect claim, anchored as it is on synergy with polymethoxylated flavonoids, is genuinely strong for the dried-leaf whole-plant preparation studied by Elfawal and colleagues, but is substantially underwritten for the traditional tea-infusion preparation actually consumed by patients in the Mueller, Blanke, and Magalhães trials reviewed in Sect. "Clinical efficacy and resistance management" [24, 25, 28, 36, 37, 48] the in vivo translation imprecision registered in Supplementary Annex F at moderate GRADE certainty under the imprecision cross-domain rating.

The principal remaining limitation is therefore the wide inter-cultivar and inter-preparation variability of artemisinin content in tea [20, 22] the inter-cultivar variability limitation cross-registered in Supplementary Annex F at low certainty under the inconsistency domain ("differential sensitivity to cultivar, harvest timing, post-harvest drying, decoction temperature–time profile"), with the per-cultivar and per-locality concentration ranges feeding the GRADE inconsistency appraisal fed back into Supplementary Annex C and Annex D.

### Clinical and public health implications

Several randomised and observational studies provide evidence for the therapeutic potential of *A. annua* preparations in falciparum malaria, although the pattern they converge on is more nuanced than a simple yes-and-no binary. Three pivotal trials in uncomplicated *P. falciparum* malaria define the upper and lower bounds. Mueller et al., 2004 Randomised parallel trial (RoB 1.0) n = 154 adults, uncomplicated *P. falciparum* Low risk of bias (Cochrane RoB 1.0) Infusion of 5 g/L or 9 g/L dried herb; 1 L/day × 7 d Day-7 cure 74% vs 91% (quinine); rapid parasite clearance in both arms; recrudescence ~ 26% by end-of-follow-up; day-28 parasitological cure not directly reported; sustained-cure extrapolation at day-28 horizon consistent with ~ 74% but not directly measured [36]. Detailed extraction of this trial's PICOS components, including sample size, allocation, follow-up windows and complete numerators/denominators, is reported in Supplementary Annex C and the methodological quality assessment per RoB 2.0 is reported in Supplementary Annex D. Blanke and colleagues subsequently compared 5 g/L infusion against sulfadoxine–pyrimethamine in semi-immune adults in the Democratic Republic of the Congo in a smaller randomised pilot series of 19 patients (10 in the *A. annua* arm and 9 in the SP arm at enrolment): day-7 cure rates were 7/10 versus 7/9, day-14 cure rates were 4/10 versus 4/9, and day-28 cure rates had collapsed to 1/9 (11.1%) in the *A. annua* arm and 3/8 (37.5%) in the SP arm, a trajectory the original authors described as a "sub-therapeutic exposure" of the plant matrix against a partially failing standard drug [24]. The complete extraction grid for this DRC pilot and its RoB 2.0 per-domain assessment are reported in Supplementary Annex C, and in Supplementary Annex D, respectively. A more recent Brazilian randomised controlled trial by Melillo de Magalhães and colleagues tested the CPQBA variety of *A. annua* (artemisinin content ≈ 1% of dried leaf) in a small cohort of patients: 4 of 7 (57.1%) of the tea-arm patients exhibited WHO Type I resistance with parasitaemia returning between day 14 and day 21, and the remaining tea-arm patients exhibited Type II or III resistance, a relapse pattern that did not establish non-inferiority to the standard artemether–lumefantrine comparator beyond 14 days of follow-up [28]. The corresponding extraction is in Supplementary Annex C, with the RoB 2.0 evaluation in Supplementary Annex D.

Two complementary studies calibrate the clinical evidence: a Ugandan community randomised trial by Ogwang and colleagues enrolled 132 flower-farm workers to once-weekly *A. annua* infusion versus placebo for 9 months and documented a 55% reduction in the risk of experiencing more than one episode of malaria (12/67 versus 26/65; p = 0.005), with bitter taste as the most commonly reported side effect and no serious adverse events [40]; and a case series from the Democratic Republic of the Congo by Daddy and colleagues described 18 patients with severe malaria refractory to both ACT and intravenous artesunate who, on compassionate grounds, received dried-leaf *A. annua* tablets and who re-cleared their parasitaemia the severely ill upper-bound counterpart to the Ogwang healthy-worker prevention case at the lower extreme [41]. The extraction grids for these two complementary studies are reported in Supplementary Annex C.

From a public-health perspective the affordability and accessibility of *A. annua* preparations continue to render them an attractive option for malaria control in resource-constrained regions of sub-Saharan Africa [7, 18]. Unlike ACTs, which face recurrent challenges related to high costs, supply-chain failures, and stock-outs at peripheral health facilities, *A. annua* can in principle be locally cultivated, prepared at household level, and administered without prescription a fact that, at a time when the World Malaria Report 2025 records a growing mismatch between biological threats and shrinking operational resources on the continent, gives the whole-plant preparation its principal decisional appeal [7, 18]. However, the absence of rigorous standardisation in preparation methods — from cultivar to leaf-to-water ratio, infusion time, and storage leads to inconsistent dosing and, as documented in §3.6, to a wide inter-cultivar range of artemisinin content in teas (0.1 → ~ 48 mg/L), with the Weathers & Towler 2012 analytical-chemistry caveat that polymethoxylated flavonoids like casticin are themselves poorly recovered from standard infusions reinforcing the problem at the matrix rather than the parent-drug level [20, 24, 28, 48]. The potential for sub-therapeutic exposure that follows from this variability raises significant concerns about the inadvertent acceleration of the resistance-selection dynamics documented in Pfkelch13-endemic African settings [18, 23, 32, 34, 38]. These tensions between local accessibility and pharmacokinetic reliability define the central public-health dilemma addressed by this systematic review. The full search strategy that identified the evidence base for this dilemma, including the four bibliographic databases plus citation searching and hand-searching of reference lists, is detailed in Supplementary Annex B, and the PRISMA 2020 reporting framework against which the §3.7 narrative was constructed is detailed in Supplementary Annex A [33].

The safety profile of *A. annua* herbal-infusion preparations is generally favourable within the doses and durations tested in the included human trials and observational studies. Reported side effects were typically mild and gastrointestinal in nature in Mueller, Blanke, and Magalhães nausea or epigastric discomfort, bitter taste noted explicitly by Ogwang and no serious adverse events were reported in the included adult or paediatric cohorts apart from one serious tea-related event recounted in literature reviews [24, 28, 36, 40]. The complete extraction of these safety signals is reported in Supplementary Annex C each row carrying the verbatim adverse-event counts and the duration of follow-up used to establish the safety conclusion. Pre-clinical in vivo data corroborate a reasonable margin of safety within the dose ranges tested in murine models; Elfawal and colleagues did not document significant whole-plant *A. annua* toxicity at the doses capable of producing parasitological cure and slowing resistance evolution in the SCID-mouse model of *P. falciparum* [25, 37]. The murine-model evidence is extracted in Supplementary Annex C. Read against the broader WHO herbal-medicine framework for traditional-use preparations, these in vivo data support the position of *A. annua* tea as a preparation whose safety is consistent with the historical-cultural-use category when consumed within documented dose ranges [18, 21]. However, a critical lack of specific safety data persists regarding excessive consumption, intermittent prophylactic dosing regimens in healthy adults, or use during pregnancy or in children under five, and explicit caution is therefore warranted in these vulnerable populations [18, 21, 28].

The resistance-surveillance landscape sharpens the public-health interpretation of these clinical results. The emergence and clonal expansion of validated Pfkelch13 propeller-domain mutations specifically R561H in Rwanda (documented from the 2013–2015 National Malaria Programme clinical efficacy trial through the 2018 + Rukara surveillance expansion) and A675V across East and Horn of African sites, with concordant multi-marker prevalence across Ugandan sites in 2023–2024 underscores the structural fragility of contemporary antimalarial efficacy against the African *P. falciparum* reservoir [8–11]. The molecular-marker extraction for these four East African surveillance studies is reported in Supplementary Annex C with the corresponding methodological quality assessment in Supplementary Annex D. A structured narrative summary of the GRADE-style certainty ratings for all twelve outcomes (parasitological clearance at day 7, day-28 cure, multiple-episode prevention, in vitro synergy, IC₅₀ reduction, resistance emergence, heme-binding affinity in mutant PfKelch13, geographical expansion of partial resistance, flavonoid recovery, cultivar variability, and overall safety) appears in Supplementary Annex F. Sustained high prevalence of multiple markers, including reduced susceptibility both to artemisinin and to its partner drugs, has been documented in northern Uganda during 2023–2024, and the broader context is one in which the World Malaria Report 2025 records that both biological threats and the financial strength of the response are diverging in the wrong direction [7, 10, 34]. The full 100-record PRISMA 2020 selection flow behind this Uganda/Rwanda/Tanzania evidence is detailed in Supplementary Annex B, with the 46 records excluded at screening plus the 10 records excluded at full-text eligibility itemised, with reasons, in Supplementary Annex E.

Integrating *A. annua* preparations into formal malaria-control programmes therefore requires strict alignment with WHO policy positions, which currently recommend against artemisinin monotherapy including herbal infusions and herbal teas in order to preserve drug longevity, and which increasingly emphasise the shift toward triple-combination therapies in regions where resistance to both the artemisinin and the partner drug is rising, in tandem with rigorous molecular surveillance of Pfkelch13, Pfmdr1, and related resistance markers [7, 16–18, 35]. The GRADE-style certainty grades for these WHO policy-alignment outcomes are reported in Supplementary Annex F, and a structured narrative summary, mechanism by mechanism, corroborating that whole-plant matrix-effect inference remains biologically plausible but only moderate-certainty in the clinical domain is reported in Supplementary Annex F under the "In vitro combination synergy" and "Resistance emergence dynamics" rows. The convergence of pre-clinical matrix-effect evidence (§3.5, §3.6) and the clinical recrudescence data surveyed in this section establishes that *A. annua* herbal tea can serve only as a regulated, supportive adjourning intervention within resistance-containment programmes and never as monotherapy in Pfkelch13-endemic settings [18, 23, 25, 35, 48].

Practically, this means that any operational use of the preparation should be paired with rigorous HPLC–MS quantification of artemisinin and the principal co-extracted polymethoxylated flavonoids, with documented daily-dose exposure rather than cup-counting, with screening for Pfkelch13 mutations at the point of care or in the affected community, and with continual pharmacovigilance in vulnerable populations [18, 20, 23, 24, 28, 35, 48]. The complete methodological framework against which this operational recommendation is structured is reported in Supplementary Annex A [33], the PROSPERO registration metadata that prospectively endorsed the present operational synthesis is reported in Supplementary Annex G, and the full alphabetical list of all abbreviations used throughout this manuscript is reproduced in the Abbreviations table at the end of the main text and cross-referenced in Supplementary Annex H for the expanded nomenclature, controlled-vocabulary mapping, and MeSH/EMA preferred-term correspondences.

## Discussion

The review synthesises six interlocking thematic strands. The first strand concerns the phytochemical mechanisms that operate beyond artemisinin in whole-plant *A. annua* preparations. While artemisinin is the most extensively characterised bioactive constituent of *A. annua*, accumulating evidence indicates that other phytochemicals notably the flavonoids quercetin, luteolin, and casticin, the non-peroxidic sesquiterpenes arteannuin B and artemisitene, and the coumarins contribute substantially to the antimalarial activity of the whole-plant matrix [26], as catalogued in Supplementary Annex C with their corresponding risk-of-bias appraisals tabulated in Supplementary Annex D under the RITAM-in-vitro appraisal framework. These secondary metabolites act in synergy with artemisinin through a polypharmacological mechanism that simultaneously engages multiple parasitic targets and elevates the genetic barrier to resistance [23, 25], a mechanistic premise retained at moderate GRADE certainty in Supplementary Annex F on the grounds of mechanistic coherence but pharmacological heterogeneity across cultivars. Several experimental studies have demonstrated that whole-plant *A. annua* preparations provide more sustained antimalarial effects than purified artemisinin administered at equivalent doses, with the secondary metabolites stabilising and potentiating the activity of the parent sesquiterpene lactone [25, 26, 37]. Suberu and colleagues reported that combining artemisinin with non-peroxidic flavonoids in vitro consistently reduced *P. falciparum* IC₅₀ values: at an artemisinin-to-flavonoid molar ratio of 1:3, combination indices were consistently below unity (CI = 0.36 for luteolin, 0.42 for quercetin, and 0.58 for casticin), and IC₅₀ values fell below 1 µmol/L for the most active combinations [23]. Rasoanaivo and colleagues documented that *A. annua* extracts combining artemisinin with co-extracted polyphenols outperformed purified artemisinin against several drug-resistant *P. falciparum* strains, an effect attributed to the redundant biological pathways targeted by the plant matrix [26]. Important caveats temper these observations. The inter-cultivar variability of secondary-metabolite profiles is substantial: HPLC–MS (high-performance liquid chromatography coupled to mass spectrometry) analyses have shown that artemisinin, dihydroartemisinic acid, and arteannuin B yields can vary by an order of magnitude across cultivars, harvest timings, and soil conditions [20, 37], with the per-cultivar concentration range (~ 0.3–15.7 mg/L across the eleven-location Beninese–Mediterranean survey) and post-harvest loss (24–48% over a single year of storage) tabulated in Supplementary Annex C. The potentiation observed in vitro has not been quantitatively reproduced in adequately powered human trials, and the matrix effect should therefore be considered biologically plausible but not yet clinically validated [25, 26]. The promising yet preliminary nature of the synergy data, together with the documented variability in extract composition, indicates that *A. annua* infusions cannot yet be standardised to a reproducible phytomedicine without upstream agronomic and phytochemical process control [23, 37].

The second strand concerns the endoperoxide-bridge chemistry that underlies artemisinin's pharmacological activation. As illustrated in Fig. 2, the pharmacophore of artemisinin is defined by an internal endoperoxide bridge (the dioxygen motif formally written as –O–O–), which is structurally conserved across the entire family of semi-synthetic derivatives in clinical use (artesunate, artemether, dihydroartemisinin) and across the sesquiterpene fraction recovered in *A. annua* infusions [19, 20]. The therapeutic activation of this pharmacophore requires intra-parasitic iron in its ferrous state: free heme (ferroprotoporphyrin IX, Fe2⁺) is released as a by-product of host haemoglobin digestion within the parasite's acidic digestive vacuole, and it is the Fe2⁺ oxidation state, not Fe3⁺, that catalyses the reductive cleavage of the bridge [19]. The acidic pH of the digestive vacuole (approximately 5.2–5.5) and the high local concentration of free heme together concentrate this reaction at the site of action, which partly explains the selectivity of artemisinin for *Plasmodium*-infected erythrocytes relative to uninfected host cells [19]. The carbon-centred radicals and the secondary reactive oxygen species generated through this cascade form covalent bonds with vital parasite proteins and with heme itself, causing widespread oxidative stress, lipid peroxidation of parasite membranes, disruption of haemoglobin-derived detoxification pathways, and ultimately parasite death [19, 23, 26]. In the modern pharmacological literature, three families of reactive intermediates are therefore distinguished carbon-centred radicals, reactive oxygen species, and alkylating intermediates and their integrated damage collectively targets the PfATP6 SERCA-type Ca2⁺-ATPase, parasite membranes, and the heme-polymerisation machinery that detoxifies free haem in the digestive vacuole [23, 26]. Rasoanaivo and colleagues further emphasise that this alkylating activity is potentiated when artemisinin is delivered in combination with flavonoids such as quercetin, luteolin, and casticin, because the latter inhibit parasite CYP3A4-mediated detoxification and prolong the residence time of artemisinin radicals at the site of action [23, 26]. Recent molecular and computational work has refined this picture: photoaffinity probe studies have identified additional artemisinin-binding targets in the intraerythrocytic developmental cycle [30], and Pfkelch13 proteins harbouring resistance-conferring propeller mutations display reduced heme-binding affinity and decreased artemisinin activation [31]. Several recent comprehensive reviews have consolidated this mechanistic substrate [32, 38, 39]. The endoperoxide-driven mechanism summarised above is mechanistically conserved across *P. falciparum*, *P. vivax*, *P. ovale*, *P. malariae*, and *P. knowlesi*, and is the chemical feature that links the modern pharmaceutical derivatives back to the empirical use of *A. annua* tea in traditional Chinese medicine [19, 20, 23].

Figure 2 proposed antiplasmodial mechanism of action of artemisinin and the matrix effect of co-extracted *Artemisia annua* phytochemicals on *Plasmodium falciparum*. Upon entry of artemisinin into the *Plasmodium*-infected erythrocyte, free heme (ferroprotoporphyrin IX) released from haemoglobin proteolysis catalyses the reductive cleavage of the endoperoxide bridge (–O–O–) of the sesquiterpene, generating a cascade of reactive oxygen-centred and carbon-centred free radicals. These radicals covalently alkylate vital parasite proteins and membranes, disrupt heme crystallisation, and trigger oxidative damage that eradicates the parasite. Co-extracted *A. annua* phytochemicals (flavonoids, non-peroxidic sesquiterpenes) contribute the matrix effect by inhibiting parasite CYP3A4-mediated detoxification and prolonging artemisinin-radical residence time, thereby slowing the evolution of Pfkelch13-mediated resistance

The third strand concerns the clinical efficacy evidence. Across the 30 retained studies, the clinical efficacy of whole-plant *Artemisia annua* infusions consistently displayed a paradoxical pattern: rapid initial parasite clearance, in many cases comparable to that of conventional antimalarials, but high day-28 recrudescence that falls well short of the PCR-adjusted cure rates documented in parallel African ACT cohorts. The earliest randomised clinical trial, by Mueller and colleagues, enrolled 154 patients with uncomplicated *P. falciparum* malaria in a low-transmission African setting and randomised them to infusions prepared from 5 g/L or 9 g/L of dried herb, with one litre consumed daily for seven days [36]. At day 7, the cure rate averaged 74% in the *A. annua* arms versus 91% in the quinine arm, and the authors concluded that "monotherapy with *Artemisia annua* L. cannot be recommended as alternative to modern antimalarials, but may deserve further investigation" because, despite the early resolution of parasitaemia and clinical symptoms, the recrudescence rate in the *A. annua* arms rose steeply over the ensuing weeks. The Mueller 2004 trial did not directly report day-28 parasitological cure as a primary endpoint; an end-of-follow-up recrudescence of approximately 26% overall is consistent with a sustained-cure extrapolation of ~ 74% at that horizon, but that figure is derived and should not be interpreted as a directly measured day-28 endpoint. The Mueller signal was corroborated and extended by Blanke and colleagues, who compared a 5 g/L *A. annua* infusion against sulfadoxine–pyrimethamine in semi-immune adults with uncomplicated falciparum malaria [24]. In that trial, the day-7 cure rate was 7/10 in the *A. annua* arm and 7/9 in the SP comparator; the day-14 cure rate declined in parallel, to 4/10 versus 4/9; and by day 28 the cure rate had collapsed to 1/9 (11.1%) in the *A. annua* arm and 3/8 (37.5%) in the SP comparator. Substituting quinine for SP does not change the central finding that whole-plant infusion, as monotherapy, cannot reliably sustain a 28-day parasitological cure in the populations studied [24, 36]. A more recent Brazilian randomised controlled trial by Melillo de Magalhães and colleagues tested a higher-artemisinin UNICAMP variety (CPQBA, with an artemisinin content of approximately 1% of dried leaf) at a dose of 1.25 g of dried leaves in 250 mL of just-boiled water consumed every six hours for seven days, yielding a cumulative artemisinin exposure of approximately 175 mg, against an artemether–lumefantrine regimen delivering approximately 525 mg of artemether [28]. Although parasitaemia in the tea group became negative in the first days of treatment despite the one-third dose, 4 of 7 patients (57.1%) treated with the *A. annua* tea exhibited WHO Type I resistance, with return of parasitaemia between day 14 and day 21, and the remaining tea-group patients exhibited Type II or III resistance. The Magalhães finding reinforces a now-consistent pattern: faster-than-expected initial clearance at sub-equivalent artemisinin doses, attributable plausibly to the contribution of flavonoids and other co-extracted phytochemicals, followed by recrudescence that the lower dose, the absence of a long-acting partner drug, or the inter-cultivar variability of the preparation cannot reliably prevent [28]. Two complementary strands of clinical evidence further calibrate the day-28 limitation. A Ugandan community randomised trial by Ogwang and colleagues, in which 132 flower-farm workers without *A. annua* exposure at baseline were allocated 1:1 to either *A. annua* infusion or placebo for 9 months of observation, found a 55% reduction in the risk of experiencing more than one episode of malaria (12/67 in the *A. annua* arm versus 26/65 in the placebo arm; *p* = 0.005), with bitter taste the most commonly reported side effect and no serious adverse events recorded [40]. A second strand, reported by Daddy and colleagues as a case series from the Democratic Republic of the Congo, described 18 patients with severe malaria who were refractory to standard ACT and to intravenous artesunate and who, on compassionate grounds, received dried-leaf *A. annua* tablets; all 18 cleared their parasitaemia [41]. Together, the prevention trial and the severe-malaria case series define the lower and upper bounds of the evidence: prophylactic benefit consistent with sub-curative dosing, and therapeutic benefit in ACT-refractory severe disease, with the mainstream uncomplicated-malaria cure deficit at day 28 cleanly bracketed between these two uses [40, 41].

The day-28 deficit of *A. annua* monotherapy becomes quantitatively sharpest when set against the modern ACT benchmarks documented in African cohorts. In the 4ABC African paediatric head-to-head study, day-28 PCR-adjusted adequate clinical and parasitological response was 95.5% for artemether–lumefantrine (1 094/1 146), 96.8% for artesunate–amodiaquine (880/909), and 97.3% for dihydroartemisinin–piperaquine (1 019/1 047) [42]. Pyronaridine–artesunate achieved a per-protocol PCR-adjusted day-28 cure rate of 98.6% (7 369/7 746) and an intention-to-treat rate of 90.9% (7 705/8 478) in the real-world SAFI cohort enrolling patients across five African countries [43], and the 2022–2023 Liberian efficacy survey reported PCR-adjusted day-28 rates of 100% and 95.9% for AL and 100% and 94.4% for ASAQ at the two study sites [44]. The day-28 cure rates of *A. annua* monotherapy reported by Blanke (11.1%) and Magalhães (none of the seven Brazilian patients sustained a day-28 cure) fall markedly below this 95.5–98.6% ACT benchmark band, an order-of-magnitude gap that frames the present-day clinical use of *A. annua* infusion as a complementary or adjourning intervention rather than a replacement for ACT [24, 28, 36, 42–44].

The fourth strand extends the mechanistic discussion to the matrix effect that emerges from co-extracted secondary metabolites. Beyond the endoperoxide-bridge mechanism, the whole-plant matrix contains a polypharmacological cocktail in which the flavonoids quercetin, luteolin, and casticin, the non-peroxidic sesquiterpenes arteannuin B and artemisitene, the coumarins exemplified by scopoletin, and a broader spectrum of polyphenols all jointly contribute antimalarial activity and resistance-modulating effects [23, 26]. In the phytotherapy literature this combination is described as plant-based artemisinin combination therapy (pACT), a structural analogue at the biological level of conventional ACT: the matrix is treated as if it were a pharmaceutical in which several molecularly distinct antimalarial elements were pre-formulated in a single dosing unit [23, 26]. Suberu and colleagues quantified the antiplasmodial activity of *A. annua* aqueous extracts and isolated artemisinin against drug-sensitive 3D7 *P. falciparum* and against chloroquine-resistant and multidrug-resistant comparator strains: the central finding is that whole-plant infusions consistently outperform the equivalent-concentration purified artemisinin by a factor of three to five in IC₅₀, indicating that the co-extracted secondary metabolites amplify parasite-killing activity beyond what artemisinin alone delivers against either sensitivity phenotype [23]. At the molecular level, Rasoanaivo and colleagues established that the polyphenols and flavonoids in *A. annua* extracts inhibit parasite CYP3A4-mediated detoxification and thereby prolong the residence time of artemisinin radicals at the parasite's digestive vacuole, an effect plausibly distributed across the redundant biological pathways of the plant matrix [26]. Beyond the headline CI numbers, Suberu and colleagues documented a detailed synergy-class breakdown: mono-caffeoylquinic acids, tri-caffeoylquinic acid, artemisinic acid, and arteannuin B showed additive interaction against the chloroquine-sensitive reference strain, while rosmarinic acid was synergistic in the chloroquine-sensitive strain but strongly antagonised artemisinin in the chloroquine-resistant strain, with arteannuin B emerging as the only co-extracted metabolite that retained synergy against the chloroquine-resistant parasites [23]. The Chou–Talalay combination-index framework captures the same trend at the level of specific flavonoid pairings: at an artemisinin-to-flavonoid molar ratio of 1:3, combination indices of 0.36 for luteolin, 0.42 for quercetin, and 0.58 for casticin have been documented in *P. falciparum* 3D7 cultures, indicating synergistic interaction by a strict pharmacological definition [23]. From a pharmacokinetic perspective, the matrix effect is biologically plausible, though the supporting evidence carries quality-control liabilities. Heide documented substantial inter-cultivar variability in artemisinin yield within traditional teas, with concentrations ranging from approximately 0.1 mg/L to nearly 48 mg/L across cultivars and harvest conditions [20], and the de Ridder review of *A. annua* as a self-reliant treatment in resource-limited settings emphasised that the matrix effect is not merely academic for endemic African populations because the combination of artemisinin with locally available co-extracted polyphenols provides a biologically defensible rationale for traditional use while simultaneously flagging the public-health risks of sub-therapeutic exposure when standardised preparation is absent [21, 35]. Pre-clinical resistance-modulation evidence gives the matrix effect its strongest quantitative leg. Elfawal and colleagues demonstrated in a murine model that dried whole-plant *A. annua* slowed the evolution of artemisinin resistance and partially overcame pre-existing resistance in Pfkelch13-mutant parasites [25, 37]. The 2015 follow-up quantified this advantage precisely: stable resistance to whole-plant therapy was achieved three times more slowly than stable resistance to an equivalent dose of pure artemisinin, and whole-plant therapy proved even more resilient than the double dose of pure artemisinin [25]. The proposed mechanistic interpretation is that the matrix elevates the genetic barrier to resistance by combining artemisinin-mediated alkylation of parasite proteins with polyphenol-mediated inhibition of *P. falciparum* detoxification pathways, multi-targeting strategies that raise the number of independent resistance mutations the parasite must accumulate to escape the treatment [23, 25, 26, 37]. Important caveats temper the translation of the matrix effect from bench to bedside. The in vitro potency ratios documented above are sensitive to cultivar, leaf-to-water ratio, decoction time, and harvest timing, with preparations lacking rigorous standardisation yielding widely divergent results [20, 23, 24, 36]. The clinical trials reviewed above did not demonstrate a clinical matrix effect on day-28 recrudescence: the reduction in early parasitaemia was comparable across *A. annua* infusions and standard therapy, but the high recrudescence rate documented particularly by Blanke (day-28 cure rate of 1/9 versus 3/8 for the sulfadoxine–pyrimethamine comparator) and Mueller (day-7 cure rate of 74% versus 91% for quinine) suggests that the in vivo concentrations of the polyphenol-to-artemisinin ratio achievable through one litre per day of 5 g/L infusion are likely to lie at the lower extreme of the in vitro dose–response curve [23, 24, 26, 28, 36]. Furthermore, the WHO categorically discourages artemisinin monotherapy including herbal brews in any form, on the grounds that sub-therapeutic exposure is one of the principal drivers of the contemporary African resistance dynamic [18, 35]. Translated into the framework of this systematic review, the matrix effect constitutes a biologically defensible and pre-clinically supported target of phytomedicine research, but not a clinical recommendation for monotherapy in resistance-endemic settings, and any operational use of whole-plant *A. annua* should be paired with rigorous HPLC–MS standardisation of artemisinin and principal flavonoid content and with molecular surveillance of Pfkelch13 and Pfmdr1 in the target population [16–18, 20, 23, 35].

The fifth strand concerns the analytical-chemistry constraints on reproducibility. Although the reductive cleavage of the endoperoxide bridge by free ferrous heme within the acidic digestive vacuole of the intra-erythrocytic parasite constitutes the central chemical step of artemisinin's pharmacology, the broader pharmacological activity of *Artemisia annua* extends well beyond artemisinin alone and is distributed, in a quantifiable manner, across a chemically diverse matrix of co-extracted secondary metabolites [23, 26]. This matrix contains the flavonoids quercetin, luteolin, and casticin; the non-peroxidic sesquiterpenes arteannuin B, artemisitene, and 9-epi-artemisinin; coumarins exemplified by scopoletin; and additional polyphenols that together comprise what Caesar and Cech describe as approximately thirty co-occurring flavonoids and sesquiterpenes, some of which possess minor antiplasmodial activities in their own right [23, 26, 49]. Flavonoids are the most extensively characterised of these non-artemisinin co-extractives at the pharmacological level: they inhibit parasite CYP3A4-mediated detoxification while their own intrinsic IC₅₀ values against *P. falciparum* 3D7 fall within the low-micromolar range an effect Ferreira and colleagues describe in the broader medicinal-chemistry literature as a "resurgent idea that multi-component drug therapy might be better than monotherapy," giving phytochemical plausibility to the matrix-effect concept [23, 26, 50]. The quantitative chemistry of the matrix has been characterised by HPLC–MS, GC–MS (gas chromatography–mass spectrometry), and NMR (nuclear magnetic resonance) fingerprinting across a range of cultivars, harvest timings, and infusion protocols, and the resulting analytical profile documents a fluctuating phytochemical fingerprint that is highly sensitive to cultivar identity, soil conditions, harvest timing, post-harvest drying, and the temperature–time profile of the decoction step [20]. Across documented traditional preparations, artemisinin content ranges from approximately 0.1 mg/L to nearly 48 mg/L, and this order-of-magnitude variation is the headline obstacle to any reproducible dose–response interpretation of the matrix [20]. Within a single controlled preparation, however, the chemical identity of the matrix becomes much more sharply defined: Suberu and colleagues quantified the principal co-extracted constituents of the *A. annua* tea preparation used in their own in vitro IC₅₀ assays as artemisinin 47.5 ± 0.8 mg/L, dihydroartemisinic acid 70.0 ± 0.3 mg/L, arteannuin B 1.3 ± 0.0 mg/L, and the polyphenolic flavonoid isovitexin 105.0 ± 7.2 mg/L, with additional polyphenolic acids recovered at lower concentrations [23]. A complementary untargeted-metabolomic survey of teas from one Mediterranean and ten Beninese cultivation sites reported a continental-scale artemisinin range of 0.3 mg/L in plants cultivated in France to 15.7 mg/L in plants cultivated in Benin, a 24–48% one-year post-harvest storage loss across three localities, and the detection of previously uncharacterised alkaloids that correlated statistically with the antiplasmodial activity of the tea preparation beyond what artemisinin content alone could explain [22]. Three distinct manufacturing modalities yield differentiated phytochemical fingerprints and therefore differentiated in vivo antiplasmodial profiles. Conventional solvent extraction with ethanol or methanol co-extracts artemisinin together with the polar flavonoid fraction and is the methodology underlying most of the IC₅₀ evidence reviewed above; maceration preserves heat-sensitive terpenes including arteannuin B and artemisitene and remains the dominant modality in resource-limited settings; and SFE-CO₂ provides a high-purity, solvent-free alternative that is considered the method of choice for high-grade pharmaceutical production [20, 21]. The same authors also documented that 9-epi-artemisinin and artemisitene themselves display mild-to-strong antagonistic interactions with artemisinin in the SYBR Green I assay despite having intrinsic IC₅₀ values below 1 µmol/L, a finding that directly calibrates the "everything in the matrix synergises with artemisinin" narrative against which the broader claim of consistency must be read [23]. A critical methodological caveat, however, constrains the generalisability of the in vitro synergy findings to traditional tea preparations. Weathers and Towler demonstrated in 2012 that under a standard hot-water infusion protocol reported to be highly effective for artemisinin extraction, only 1.8% of casticin and essentially undetectable artemetin were recovered from the infusion, and that 40% of the casticin that was recovered was lost after 24 h at room temperature [48]. In a parallel analytical study, the Elfawal group quantified that a 5-min boiling water extraction recovers approximately 90% of the plant's artemisinin but loses approximately 99% of some of the original flavonoids that reportedly synergise with artemisinin, a finding that led the same authors to formally distinguish the whole-plant dried-leaf preparation they were testing from any tea or infusion preparation [37]. Read together, these two analytical-chemistry findings establish that the matrix-effect claim, anchored as it is on synergy with polymethoxylated flavonoids, is genuinely strong for the dried-leaf whole-plant preparation studied by Elfawal and colleagues, but is substantially underwritten for the traditional tea-infusion preparation actually consumed by patients in the Mueller, Blanke, and Magalhães trials [24, 25, 28, 36, 37, 48].

The sixth strand addresses the public-health implications in Pfkelch13-endemic African settings. From a public-health perspective the affordability and accessibility of *A. annua* preparations continue to render them an attractive option for malaria control in resource-constrained regions of sub-Saharan Africa [7, 18]. Unlike ACTs, which face recurrent challenges related to high costs, supply-chain failures, and stock-outs at peripheral health facilities, *A. annua* can in principle be locally cultivated, prepared at household level, and administered without prescription a fact that, at a time when the World Malaria Report 2025 records a growing mismatch between biological threats and shrinking operational resources on the continent, gives the whole-plant preparation its principal decisional appeal [7, 18]. However, the absence of rigorous standardisation in preparation methods from cultivar to leaf-to-water ratio, infusion time, and storage leads to inconsistent dosing and, as documented above, to a wide inter-cultivar range of artemisinin content in teas (0.1– ~ 48 mg/L), with the Weathers & Towler 2012 analytical-chemistry caveat that polymethoxylated flavonoids like casticin are themselves poorly recovered from standard infusions reinforcing the problem at the matrix rather than the parent-drug level [20, 24, 28, 48]. The potential for sub-therapeutic exposure that follows from this variability raises significant concerns about the inadvertent acceleration of the resistance-selection dynamics documented in Pfkelch13-endemic African settings [18, 23, 32, 34, 38]. These tensions between local accessibility and pharmacokinetic reliability define the central public-health dilemma addressed by this systematic review. The safety profile of *A. annua* herbal-infusion preparations is generally favourable within the doses and durations tested in the included human trials and observational studies. Reported side effects were typically mild and gastrointestinal in nature in Mueller, Blanke, and Magalhães (nausea or epigastric discomfort, with bitter taste noted explicitly by Ogwang), and no serious adverse events were reported in the included adult or paediatric cohorts apart from one serious tea-related event recounted in literature reviews [24, 28, 36, 40]. Pre-clinical in vivo data corroborate a reasonable margin of safety within the dose ranges tested in murine models; Elfawal and colleagues did not document significant whole-plant *A. annua* toxicity at the doses capable of producing parasitological cure and slowing resistance evolution in the SCID-mouse model of *P. falciparum* [25, 37]. Read against the broader WHO herbal-medicine framework for traditional-use preparations, these in vivo data support the position of *A. annua* tea as a preparation whose safety is consistent with the historical-cultural-use category when consumed within documented dose ranges [18, 21]. A critical lack of specific safety data persists, however, regarding excessive consumption, intermittent prophylactic dosing regimens in healthy adults, or use during pregnancy or in children under five, and explicit caution is therefore warranted in these vulnerable populations [18, 21, 28]. The resistance-surveillance landscape sharpens the public-health interpretation of these clinical results. The emergence and clonal expansion of validated Pfkelch13 propeller-domain mutations specifically R561H in Rwanda (documented from the 2013–2015 National Malaria Programme clinical efficacy trial through the 2018 + Rukara surveillance expansion) and A675V across East and Horn of African sites, with concordant multi-marker prevalence across Ugandan sites in 2023–2024 underscores the structural fragility of contemporary antimalarial efficacy against the African *P. falciparum* reservoir [8–11]. Sustained high prevalence of multiple markers, including reduced susceptibility both to artemisinin and to its partner drugs, has been documented in northern Uganda during 2023–2024, and the broader context is one in which the World Malaria Report 2025 records that both biological threats and the financial strength of the response are diverging in the wrong direction [7, 10, 34]. Integrating *A. annua* preparations into formal malaria-control programmes therefore requires strict alignment with WHO policy positions, which currently recommend against artemisinin monotherapy, including herbal infusions and herbal teas, in order to preserve drug longevity, and which increasingly emphasise the shift toward triple-combination therapies in regions where resistance to both the artemisinin and the partner drug is rising, in tandem with rigorous molecular surveillance of Pfkelch13, Pfmdr1, and related resistance markers [7, 16–18, 35]. The convergence of pre-clinical matrix-effect evidence and the clinical recrudescence data surveyed in this section establishes that *A. annua* herbal tea can serve only as a regulated, supportive adjourning intervention within resistance-containment programmes—and never as monotherapy in Pfkelch13-endemic settings [18, 23, 25, 35, 48]. The complete methodological framework against which this operational recommendation is structured is reported in Supplementary Annex A [33], the PROSPERO registration metadata that prospectively endorsed the present operational synthesis is reported in Supplementary Annex G, and the full alphabetical list of all abbreviations used throughout this manuscript is reproduced in the Abbreviations table at the end of the main text and cross-referenced in Supplementary Annex H.

## Conclusions

The present systematic review synthesised twenty-five years of pre-clinical and clinical evidence on whole-plant *Artemisia annua* herbal teas across drug-sensitive, multidrug-resistant, and Pfkelch13-mutant contexts of uncomplicated *Plasmodium falciparum* malaria. Four interconnected domains were delineated: clinical and parasitological efficacy; the phytochemical matrix effect beyond artemisinin monotherapy; multi-target resistance modulation under Pfkelch13 pressure; and the safety profile across vulnerable populations. From these four domains, five principal conclusions emerge, each anchored on the results reported in §3 and pointing to an actionable implication drawn directly from those results.

First, the *A. annua* infusion delivers reproducible initial parasite clearance within 72 h across the four retained randomised trials. This clearance signal confirmed across Mueller's Rungwe trial, Blanke's DRC pilot, Magalhães's Brazilian CPQBA cohort, and Ogwang's Ugandan community study is mechanistically attributable to the reductive cleavage of the artemisinin endoperoxide bridge by ferrous heme in the parasite's digestive vacuole, as documented in Sect. "Mechanism of action: role of the endoperoxide bridge". Implication drawn from this finding: in Pfkelch13-endemic settings, where artemisinin-based combination therapy attrition is accelerating, this 72-h clearance capacity constitutes a pharmacologically defensible triage window during which parasitologically confirmed patients can be transferred to artemisinin-based combination therapy or triage-managed at community level while molecular resistance surveillance is initiated.

Second, where day-28 parasitological cure is directly reported, rates of *A. annua* monotherapy remain consistently below the artemisinin-based combination therapy benchmark band: 11.1% for Blanke (1/9) and 0% sustained day-28 cure for the seven Brazilian patients in the Magalhães arm, against 95.5–98.6% combined across the four African ACT regimens artemether–lumefantrine 95.5%, artesunate–amodiaquine 96.8%, dihydroartemisinin–piperaquine 97.3%, and pyronaridine–artesunate 98.6% [42–44]. The Mueller 2004 trial did not report day-28 cure as a primary endpoint and is excluded from this strict head-to-head figure; its documented end-of-follow-up recrudescence of approximately 26% overall [36] is consistent with a sustained-cure extrapolation of approximately 74% at that horizon, but this figure is derived and should not be interpreted as a directly measured day-28 endpoint.

Third, the matrix effect quantified in Sects. "Phytochemical synergy and the"matrix effect"" and"Phytochemistry and antiplasmodial activity" by Chou–Talalay combination indices (0.36 luteolin, 0.42 quercetin, 0.58 casticin at artemisinin-to-flavonoid 1:3) and by the three- to five-fold lower IC₅₀ of whole-plant extract relative to purified artemisinin partially offsets the reduced artemisinin activation imposed by Pfkelch13 propeller mutations. The murine *P. falciparum* model showed stable resistance emerging three times more slowly under whole-plant therapy than under equivalent-dose pure dihydroartemisinin, even in Pfkelch13-mutant lines. Implication drawn from this finding: the pre-clinical resistance-retardation signal is strong enough to justify, in endemic regions, standardised HPLC–MS–anchored whole-plant preparations rather than purified artemisinin monotherapies, provided that the inter-cultivar variability documented across studies (artemisinin concentrations between 0.1 mg/L and nearly 48 mg/L) is brought under reproducible batch-level control.

Fourth, the safety profile documented in Sect. "Clinical and public health implications" is generally favourable in healthy adults: bitter taste was the predominant side effect in Ogwang's prevention trial, gastrointestinal discomfort featured in Mueller, Blanke, and Magalhães, and no serious adverse events were reported in any retained study. However, critical evidence gaps persist for children under five, pregnant or lactating women, and patients with hepatic or renal co-morbidity. The transplacental and paediatric pharmacokinetics of artemisinin delivered through infusion rather than as a standardised pharmaceutical remain essentially uncharacterised. Implication drawn from this finding: any operational integration of *A. annua* preparations into malaria control programmes in endemic African settings must explicitly exclude vulnerable populations until dedicated randomised controlled trials are conducted.

Fifth, the quality-control gap exposed by the heterogeneity of artemisinin and polymethoxylated-flavonoid recovery only 1.8% of casticin and essentially undetectable artemetin in a standard hot-water infusion, with 40% loss of recovered casticin within 24 h at room temperature is the principal determinant of sub-therapeutic exposure and, consequently, of inadvertent Pfkelch13 resistance selection. Implication drawn from this finding: deployment of *A. annua* infusions must be paired with mandatory batch-level HPLC–MS quantification of artemisinin and co-extracted flavonoids, with population-level molecular surveillance of Pfkelch13 and Pfmdr1, and with rapid pharmacovigilance for vulnerable groups.

Taken together, these five conclusions delineate a coherent translational agenda. Whole-plant *A. annua* preparations are best positioned as a regulated, pre-clinical-trials-validated complementary intervention within structured resistance-containment programmes reserved for use under pharmacovigilance, in geographic areas with confirmed Pfkelch13 circulation, in populations for whom artemisinin-based combination therapy is available but supply-chain unreliability is documented, and never as monotherapy in uncomplicated falciparum malaria. Artemether–lumefantrine, artesunate–amodiaquine, and dihydroartemisinin–piperaquine remain the cornerstone of malaria case management; *A. annua* infusions occupy an adjunctive not substitutive position whose value derives from the multi-target resistance-modulating activity of the whole-plant matrix rather than from any displacement of standard therapy.

### Four operational recommendations support this conclusion

Clinical trial reform. Future research must prioritise rigorously designed, randomised, double-blind trials using artemisinin-based combination therapy comparators with extended follow-up periods of at least 28 days to accurately detect recrudescence and to evaluate both clinical and parasitological endpoints. Trial methodology should explicitly integrate molecular surveillance of Pfkelch13 and Pfmdr1 markers alongside clinical endpoints, drawing on the harmonised framework articulated by the WHO and by the Talisuna surveillance blueprint. Because the present review detected no adequately powered head-to-head comparison of whole-plant *A. annua* infusions against artemisinin-based combination therapy in Pfkelch13-endemic populations across the retained literature, this evidence gap is the single most important priority for the next decade.

Preparation standardisation anchored on HPLC–MS quantification. To ensure reproducibility, infusion preparation methods including leaf-to-water ratios (5 g/L in the Blanke and Mueller protocols versus 9 g/L in the Mueller high-dose arm) and decoction times must be standardised. Such methods must be anchored on quantitative HPLC–MS determination of artemisinin and principal co-extracted flavonoids. With artemisinin concentrations in traditional teas fluctuating between 0.1 mg/L and nearly 48 mg/L across cultivars and harvest conditions, quantification prior to clinical dosing is non-negotiable.

Evidence-based dosing in vulnerable populations. Dosing regimens must be established for demographically diverse populations, with explicit prioritisation of high-risk groups such as children under five, pregnant women, and breastfeeding mothers populations for which the present review identified a critical lack of safety and efficacy data. Parallel investigations into potential drug-herb interactions should be undertaken to ensure no antagonism against partner artemisinin-based combination therapy drugs and to maintain compatibility with intermittent preventive treatment in pregnancy protocols. The WHO herbal-medicine framework for traditional-use preparations may serve as the regulatory anchor for these protocols.

Molecular resistance surveillance and community engagement. The systematic documentation of safety and tolerability must be coupled with continuous molecular surveillance of Pfkelch13 (especially the R561H, A675V, and other propeller-domain mutations now confirmed in Rwanda and Uganda) and of Pfmdr1 resistance loci. This surveillance must be population-wide and longitudinal, with cross-border data sharing under the umbrella of the WHO Global Malaria Programme, and must extend to rapid detection of putative artefactual treatment failures attributable to sub-therapeutic *A. annua* exposures that mask underlying Pfkelch13 partial resistance.

The supplementary annexes accompanying this manuscript are submitted as individual Word documents (.docx) and are referenced throughout the main text as Supplementary Annex A (PRISMA 2020 reporting checklist, 27 items), Supplementary Annex B (database-specific search strategies, four-table section), Supplementary Annex C (per-study data extraction grid, 32 rows), Supplementary Annex D (per-study risk-of-bias appraisals, Cochrane RoB 2.0 + Newcastle–Ottawa + RITAM), Supplementary Annex E (excluded-studies ledger: 46 screening-level + 10 eligibility-level), Supplementary Annex F (structured GRADE narrative synthesis across 12 pre-specified outcomes, with per-domain certainty annotations), Supplementary Annex G, and Supplementary Annex H (controlled vocabulary, MeSH/EMA preferred-term correspondences, and expanded nomenclature for the abbreviations used in the manuscript).

## Supplementary Information

Supplementary Material 1

## Data Availability

The data extracted and analyzed during this study are included within this article. Detailed data extraction forms and the study protocol are publicly available on the PROSPERO platform under the registration number **CRD420251128231**.

## Abbreviations

ACT: Artemisinin-based combination therapy
ADME: Absorption–distribution–metabolism–excretion
AJOL: African Journals Online
AL: Artemether–lumefantrine
AS: Artesunate
ASAQ: Artesunate–amodiaquine
AUC: Area under the curve
CDC: Centers for Disease Control and Prevention
CI: Combination index
Cmax: Maximum plasma concentration
ConPhyMP: Consortium for Phytotherapeutic Manufacturing Practices
CQ: Chloroquine
CYP3A4: Cytochrome P450 isoform 3A4
DHA: Dihydroartemisinin
DHA-PPQ: Dihydroartemisinin–piperaquine
DNA: Deoxyribonucleic acid
ED50: Median effective dose (50%)
EU: European Union
FDA: Food and Drug Administration
GC–MS: Gas chromatography–mass spectrometry
GMP: Good manufacturing practice
GRADE: Grading of Recommendations, Assessment, Development and Evaluation
H2O2: Hydrogen peroxide
HPLC: High-performance liquid chromatography
HPLC–MS: High-performance liquid chromatography–mass spectrometry
HRP-2: Histidine-rich protein-2
HRP-3: Histidine-rich protein-3
IC_50_: Half-maximal inhibitory concentration
iNOS: Inducible nitric oxide synthase
JP/NMR: Joint phytochemistry/nuclear magnetic resonance confirmation
LC50: Median lethal concentration
LD50: Median lethal dose (50%)
LILACS: Latin American and Caribbean Health Sciences Literature
LMIC: Low- and middle-income country
LOD: Limit of detection
LOQ: Limit of quantification
MoA: Mechanism of action
NHS: National Health Service
NOS: Newcastle–Ottawa Scale
*OR*: Odds ratio
PA: Pyronaridine–artesunate
PCR: Polymerase chain reaction
PfATP6: *Plasmodium falciparum sarcoplasmic/endoplasmic reticulum Ca2⁺-ATPase*
Pfkelch13: *Plasmodium falciparum* Kelch-13 propeller
Pfmdr1: *Plasmodium falciparum* Multidrug-resistance transporter 1
PQ: Primaquine
PRISMA: Preferred Reporting Items for Systematic Reviews and Meta-Analyses
PROSPERO: International Prospective Register of Systematic Reviews
QC: Quality control
RCT: Randomised controlled trial
RDT: Rapid diagnostic test
RITAM: RESEARCH Initiative on Traditional Antimalarial Methods
RNA: Ribonucleic acid
RoB 2.0: Revised Cochrane Risk-of-Bias Tool, version 2.0
*RR*: Risk ratio
SAFI: Surveillance of Antimalarial Fixed-dose combinations safety, Francophone initiatives
SCID: Severe combined immunodeficiency (mouse)
SFE-CO2: Supercritical fluid extraction with carbon dioxide
SOP: Standard operating procedure
SP: Sulfadoxine–pyrimethamine
t½: Elimination half-life (italic t)
UNICAMP CPQBA: Universidade Estadual de Campinas, Centro Pluridisciplinar de Pesquisas Químicas, Biológicas e Agrícolas
USA: United States of America
UV: Ultraviolet (spectrophotometric detection)
WHO: World Health Organization

## Declarations

### Role of AI application in this manuscript

During the preparation of this manuscript, the lead author used a generative-AI writing-assistant platform for: (i) English-language proofreading; (ii) creation of figures (Fig. 2 mechanism-of-action schematic) consistent with the Infectious Diseases of Poverty submission guidelines; and (iii) reference-management support.
