# Supplementary material for: Phytochemical synergy in artemisia annua herbal tea against malaria: a systematic review of its efficacy and safety in the context of emerging Pfkelch13 resistance
Source: Infect Dis Poverty. 2026 Aug 13;15:87. doi: 10.1186/s40249-026-01486-x (PMC13471599; doi:10.1186/s40249-026-01486-x)
Supplement: Supplementary file 1 [file 40249_2026_1486_MOESM1_ESM.docx]

# Phytochemical Synergy in Artemisia annua Herbal Tea against Malaria : A Systematic Review of its Efficacy and Safety in the Context of Emerging Pfkelch13 Resistance

**Jérôme Munyangi wa Nkola 1,2*, Pierre Akilimali Zalagile ^3,4^, Hendrick Lukuke Mbutshu ^5^, Spartacus Kabala Munyemo ^6^, Imani Ramazani Bin Eradi ^7,8^, Alioune Camara 9,10**

## Annex A PRISMA 2020 Checklist (27 items + sub-items)

| Section / Item | # | PRISMA 2020 Requirement | Manuscript Page / Section | Justifying element |
| --- | --- | --- | --- | --- |
| **TITLE** |  |  |  |  |
| Title | 1 | Identify the publication as a systematic review, meta-analysis, or both | Title page | "Phytochemical Synergy in Artemisia annua Herbal Tea against Malaria: **A Systematic Review**…" |
| **STRUCTURED ABSTRACT** |  |  |  |  |
| Structured abstract | 2 | Provide a structured abstract including: background, objectives, data sources, eligibility criteria, methods of assessment and synthesis, results, conclusions | Abstract | Background / Methods / Results / Conclusions sections duly filled |
| **INTRODUCTION** |  |  |  |  |
| Rationale | 3a | Describe the rationale for the review in the context of existing knowledge | §1 Background (paragraphs 1–3) | 240 million cases / 600 000 deaths annually; Pfkelch13 documented in 6 African countries (1–7) |
| Objectives | 3b | State the questions covered by the review with reference to participants, interventions, comparators, outcomes, and study design | §2.1 + PICOS box | Population, Intervention, Comparator, Outcomes, Study design specified and reported in Annex A |
| **METHODS** |  |  |  |  |
| Protocol and registration | 4a | Indicate whether a protocol exists; provide it if accessible; provide registration information (register name, number) | §2, opening | PROSPERO CRD420251128231, dated 25 November 2025, updated 15 December 2025; public URL reported in **Annex G** |
|  | 4b | Mention any modifications from the registered information | §2 + Annex G | No deviation from the protocol |
| Eligibility criteria | 5 | Specify eligibility criteria; report in full in Annex C | §2.1 + Annex C columns 1–8 | Population ≥ 6 months; intervention: standardised infusion / decoction; comparators: ACT / quinine / SP / chloroquine / purified artemisinin; outcomes: clearance days 3/7/14/28, recrudescence, IC₅₀, CI, pharmacokinetics; eligible designs |
| Information sources | 6 | Describe information sources (databases, last-search dates, filters) | §2.2 + Annex B Table B.1 | PubMed, ScienceDirect, Cochrane Library, Google Scholar (citation-tracking), Cairn.info, WHO Global Malaria Programme; last search: December 2025; languages: EN, FR, DE, ES, PT, ZH, SW |
| Search strategy | 7 | Present the full search strategy for at least one database | §2.2 + Annex B | 4 Boolean clusters: A. A. annua/qinghao/sweet wormwood; B. infusion/decoction/tea/phytomedicine; C. malaria/P. falciparum; D. efficacy/safety/synergy/resistance |
| Study selection | 8 | Specify methods used to determine eligibility; double screening, divergence management | §2.3 | Covidence, Cohen κ = 0.83; consensus, then senior arbitrator |
| Extraction process | 9 | Describe extraction methods (pilot, double extraction, autonomy) | §2.3 + Annex C | 32 extraction lines; PICOS per study; currency, cultivar, leaf/water ratio, decoction time, artemisinin per litre |
| Risk of bias assessment | 10a | Specify methods used to assess risk of bias of each included study | §2.4 + Annex D | RoB 2.0 (trials) + Newcastle–Ottawa (observational) + RITAM (phytomedicine) |
|  | 10b | Indicate whether assessment was performed at study-level or outcome-level | §2.4 | Study-level for 21 records; GRADE at outcome-level for the 12 outcomes |
| Outcome measures | 11 | Specify all result measures (direction of benefit, units, scale) | §2.1 + Abstract | Day 3/7/14/28 clearance; IC₅₀; CI (Chou–Talalay); Cmax, AUC₀₋₂₄h, AUC₀₋∞, t½; Pfkelch13 markers |
| Synthesis methods | 13a | Describe synthesis methods (tabulation, meta-analysis, GRADE) | §2.5 + Annex F | Narrative qualitative synthesis; GRADE narrative (no meta-analysis) |
|  | 13b | Describe methods of exploring heterogeneity | §2.5 | 5 g/L vs 9 g/L preparations; 5–30 min decoction; cultivar dependence; heterogeneous outcomes |
|  | 13c | Describe any sensitivity analyses | §2.5 | Stratification RoB 2.0 × IC₅₀ direction, without meta-analysis |
|  | 13d | State how publication bias was addressed | §2.4 | GRADE domains for publication bias, 12 outcomes |
| **RESULTS** |  |  |  |  |
| Study selection | 16a | Describe results of selection process (n identified, screened, eligible, included) | §3.1 + Figure 1 | n=130 → 14 duplicates removed → 86 records title-abstract screened → 46 excluded → 40 full texts assessed → 10 excluded → 30 included |
|  | 16b | Cite studies that appeared eligible but were excluded with reason | Annex E | 46 exclusions at screening (category) + 10 exclusions at eligibility (E1–E10) |
|  | 16c | Provide a PRISMA flow diagram | Figure 1 | Reproduced on results page |
| Study characteristics | 17 | Cite each included study and present its characteristics | §3 + Annex C | Table 1 (8 pivotal studies) + Annex C (32 rows) |
| Risk of bias of included studies | 18 | Present risk-of-bias assessments for each included study | Annex D | D1–D5 + global judgement for 21 records |
| Results of individual studies | 19 | For all outcomes, present per-study summarised data | §3.4–3.7 + Annex C | Mueller day-7 74% vs 91%; Blanke day-28 1/9 vs 3/8; Magalhães 0/7 |
| Synthesis of results | 20a | Summarise each outcome with indicators of certainty | §3 + Annex F | GRADE narrative: ⊕⊕⊕⊕ to ⊕⊕⊖⊖ |
|  | 20b | Present results of any meta-analysis conducted | §2.5 | No meta-analysis conducted |
|  | 20c | Describe direction of benefit and effect size if meta-analysis conducted | §2.5 | Not applicable |
|  | 20d | If survival-analysis meta-analysis conducted, describe Kaplan–Meier estimation | §3.4 | Not applicable |
| **DISCUSSION** |  |  |  |  |
| Synthesis of evidence | 24a | Summarise main results including strength of evidence for each main outcome | §3 + Conclusion 1–5 | 72 h reproducible clearance; day-28 deficit; IC₅₀ 3–5×; resistance 3× slower; favourable safety |
|  | 24b | Discuss limitations of the evidence included in the review | §3.4–3.7 limits | Inter-cultivar variability 0.1 → 48 mg/L; infusion matrix under-financed; no Pfkelch13-endemic human trial |
|  | 24c | Discuss limitations of the review methods | §2.5 | No meta-analysis; narrative synthesis |
| Interpretation | 25a | Provide an overall interpretation of results in relation to other reviews | §3.5 + Conclusion 3 | Consistent with Mueller, Elfawal, Suberu; divergent from Blanke on day-28 |
|  | 25b | Discuss implications for practice, policy and future research | Conclusion 4 recommendations | Reform of clinical trials; HPLC-MS standardisation; vulnerable population; molecular surveillance |
| **OTHER INFORMATION** |  |  |  |  |
| Registration | 24 | Provide register, number, and date | §2 + Annex G | PROSPERO CRD420251128231 |
| Conflicts of interest | 26 | Declare any conflicts of interest | Declarations | No financial or commercial conflicts |
| Funding sources | 27 | Indicate funding sources for the review | Declarations | No external funding |

## Annex B Search Strategy by Database (4 tables)

### Table B.1 Databases queried, dates, filters applied, number of records identified

| Database | Last-search date | Filters applied | Records identified | Duplicates removed |
| --- | --- | --- | --- | --- |
| PubMed | 12 December 2025 | Species: Homo sapiens; type: article + review; languages: EN, FR | 32 | 8 |
| ScienceDirect | 12 December 2025 | Year: 2000–2025; type: research article + review | 24 | 4 |
| Cochrane Library | 12 December 2025 | Type: Trials + Cochrane Reviews | 14 | 2 |
| Google Scholar (citation-tracking) | 14 December 2025 | Sort by relevance; top-100 results | 18 | — |
| Cairn.info | 12 December 2025 | Sections "Health sciences"; period 2000–2025 | 0 | — |
| WHO Global Malaria Programme | 14 December 2025 | Region: AFRO + Global; policy & surveillance documents | 12 | — |
| Citation-tracking + hand-search | 14–22 December 2025 | Reference lists of Talisuna 2012; Rosenthal 2024; Watson 2024; Harrison 2026; Schreidah 2024 | 30 | — |
| **TOTAL** | — | — | **130** | **14** |

### Table B.2 Combined Boolean architecture (4 semantic clusters)

| Cluster | Combined keywords |
| --- | --- |
| C1 — Plant | Artemisia annua OR qinghao OR sweet wormwood OR qing hao su |
| C2 — Preparation | infusion OR decoction OR tea OR "herbal preparation" OR phytomedicine OR "dried leaves" |
| C3 — Pathology | malaria OR Plasmodium falciparum OR "uncomplicated malaria" OR falciparum |
| C4 — Outcomes measured | efficacy OR safety OR pharmacokinetics OR synergy OR resistance OR "antiplasmodial" |

**Overall Boolean operators:** (C1) AND (C2) AND (C3) AND (C4); linguistic restriction ``; temporal restriction 2000–2025.

### Table B.3 Complete PubMed query

```
("Artemisia"[MeSH] OR "Artemisia annua"[tiab] OR "qinghao"[tiab]
 OR "sweet wormwood"[tiab])
AND
("Plant Preparations"[MeSH] OR "Phytotherapy"[MeSH]
 OR "infusion"[tiab] OR "tea"[tiab] OR "decoction"[tiab]
 OR "herbal preparation"[tiab])
AND
("Malaria"[MeSH] OR "Plasmodium falciparum"[MeSH]
 OR "Falciparum"[tiab] OR "uncomplicated malaria"[tiab])
AND
("Drug Synergism"[MeSH] OR "Drug Resistance"[MeSH]
 OR "Treatment Outcome"[MeSH] OR "Safety"[MeSH]
 OR efficacy[tiab] OR synergy[tiab] OR resistance[tiab])
AND
("2000/01/01"[PDAT] : "2025/12/31"[PDAT])
AND (English[lang] OR French[lang])
AND (humans[Filter] OR mice[Filter])
```

### Table B.4 Equivalent strategies for ScienceDirect, Cochrane, Cairn.info

#### ScienceDirect

TITLE-ABS-KEY("Artemisia annua" OR qinghao) AND TITLE-ABS-KEY(infusion OR tea OR decoction) AND TITLE-ABS-KEY(malaria OR "Plasmodium falciparum") AND TITLE-ABS-KEY(synergy OR resistance OR efficacy) AND PUBYEAR > 1999 AND PUBYEAR < 2026 AND (LIMIT TO (PUB, "Article" OR "Review"))

#### Cochrane Library

("Artemisia annua" OR qinghao) AND (tea OR infusion OR decoction) AND (malaria OR falciparum) AND (synergy OR efficacy OR resistance) in Trials + Cochrane Reviews 2000–2025

#### Cairn.info

("Artemisia annua" OR qinghao) ET (infusion OR tisane OR décoction) ET (paludisme OU falciparum)

Result: n=0 records retained

## Annex C Per-Study Extraction Grid (32 lines: row #1 PRISMA 2020 norm, rows #2–#32 = included records)

Column headers: # | Study (author, year) | Design | Country / setting | Population (n, characteristics) | A. annua cultivar | Leaf/water ratio; decoction time; artemisinin mg/L | Comparator arm | Primary outcome | n per arm (randomisation) | Follow-up (days) | Pfkelch13 mutational domain | Adverse events | Funding source

| # | Author, year | Design | Country | n | Cultivar | Preparation | Comparator | Primary outcome | RoB |
| --- | --- | --- | --- | --- | --- | --- | --- | --- | --- |
| 1 | Page et al., 2021 (8) | PRISMA 2020 norm | — | — | — | — | — | Methodological reference | — |
| 2 | Mueller et al., 2004 (9) | Randomised parallel trial (RoB 1.0) | Tanzania | 154 adults | Not specified | Infusion 5 g/L or 9 g/L; 1 L/d × 7 d; variable artemisinin | Quinine 10 mg/kg × 7 d | Day-7 cure 74% vs 91% | Low risk (RoB 1.0) |
| 3 | Blanke et al., 2008 (10) | Randomised double-blind trial | DRC | 19 semi-immune adults | Local | Infusion 5 g/L; 1 L/d × 7 d | Sulfadoxine–pyrimethamine | Day-28 cure 1/9 (11.1%) vs 3/8 (37.5%) | Low risk (RoB 1.0) |
| 4 | Melillo de Magalhães et al., 2016 (11) | Open-label RCT | Brazil | 53 adults | UNICAMP-CPQBA (~1% art.) | 1.25 g / 250 mL; 1 dose/6 h × 7 d ≈ 175 mg cumulative | Artemether–lumefantrine (≈525 mg cumulative) | Day-1 parasitaemia-negative; 4/7 WHO Type I resistant day-14-21 | Some concerns (RoB 2.0) |
| 5 | Ogwang et al., 2012 (12) | Cluster-randomised trial | Uganda | 132 (67/65) | Local | Weekly infusion for 9 months | Placebo | Multiple episodes: 12/67 vs 26/65 (p = 0.005) | Moderate (RoB 2.0) |
| 6 | Daddy et al., 2017 (13) | Case series | DRC | 18 (severe malaria) | Local | Dried-leaf tablets; compassionate dose | — | 18/18 clearance | High (case series) |
| 7 | Suberu et al., 2013 (14) | In vitro (3D7 + Dd2 + W2) | United Kingdom | 9 molar ratios | Cultivated A. annua | Aqueous extract vs purified artemisinin | — (inter-metabolite synergy) | IC₅₀ ratio 3–5×; CI luteolin 0.36; quercetin 0.42; casticin 0.58 | Excellent |
| 8 | Rasoanaivo et al., 2011 (15) | Mechanistic review | Madagascar/multiple | — | Multiple | Whole extracts vs compounds | — | CYP3A4 inhibition; matrix effect | Narrative review |
| 9 | Elfawal et al., 2012 (16) | In vivo (SCID mice, P. falciparum) | United States | — | Diverse | Dried leaves vs purified artemisinin (equivalent dose) | — | Whole-plant superiority in vivo | High |
| 10 | Elfawal et al., 2015 (17) | In vivo (SCID mice, Pfkelch13 mutant lines) | United States | 3 arms | Diverse | Equivalent-dose whole-plant vs DHA; double-dose pure DHA control | — | Resistance 3× slower | High |
| 11 | De Ridder et al., 2008 (18) | Review | Netherlands | — | — | — | — | African self-reliance argument | — |
| 12 | Heide, 2006 (19) | Analytical | Germany | 11 cultivars | Multiple cultivars | Standardised decoction; HPLC-MS | — | 0.1 → 48 mg/L artemisinin | High (analytical) |
| 13 | Weathers & Towler, 2012 (20) | Analytical | United States | — | — | Hot-water infusion | — | Casticin 1.8%; artemetin undetectable | High |
| 14 | Wang, 2019 (21) | Phytochemical review | China | — | — | — | — | Scopoletin, arteannuin B, artemisinic acid | — |
| 15 | Ferreira et al., 2010 (22) | Flavonoid review | United States | — | — | — | — | Antioxidants + artemisinin synergy | — |
| 16 | Aubouy et al., 2025 (23) | Untargeted metabolomics | Benin + Mediterranean | 11 sites | Local cultivars | Traditional teas | — | 0.3 → 15.7 mg/L; 24–48% loss at 1 year | High |
| 17 | Oladipo & Ojumu, 2025 (24) | SFE-CO₂ review | Nigeria | — | — | Supercritical extraction | — | High purity; terpene preservation | — |
| 18 | Gao et al., 2024 (25) | Mechanism | — | — | — | Photoaffinity probe | — | Additional intra-erythrocytic cycle targets | — |
| 19 | Rahman et al., 2024 (26) | Pfkelch13 mutant proteins | — | — | — | Heme affinity | — | Reduced affinity; decreased activation | High |
| 20 | Schreidah et al., 2024 (1) | Molecular surveillance | Rwanda | 99 patients | — | — | — | Emerging R662I; HRP2/3 del absent | High |
| 21 | Uwimana et al., 2020 (2) | Molecular surveillance | Rwanda | 138 isolates | — | — | — | R561H clonal propagation | High |
| 22 | Ishengoma et al., 2024 (4) | Molecular + clinical surveillance | Tanzania | 248 patients | — | — | AL/ASAQ | Kelch13 markers detected | High |
| 23 | Tumwebaze et al., 2022 (5) | Surveillance | Uganda (N) | 163 patients | — | — | AL | Reduced DHA + lumefantrine susceptibility | High |
| 24 | Katairo et al., 2026 (3) | Surveillance | Uganda | 391 patients | — | — | — | Multiple marker prevalence 2023-2024 | High |
| 25 | Meier-Scherling et al., 2024 (6) | Spatio-temporal modelling | Uganda | — | — | — | — | Selection rate comparable to Southeast Asia | High |
| 26 | Schmedes et al., 2021 (27) | 9-country surveillance | 9 African countries | 1117 isolates | — | — | — | Pfkelch13 mutations confirmed 2014-2018 | High |
| 27 | van Loon et al., 2025 (28) | Surveillance | Uganda | 92 patients | — | — | — | Kelch13 markers 2024 | High |
| 28 | Assefa et al., 2024 (7) | Regional synthesis | Horn of Africa / E Africa | — | — | — | — | Risk framework | — |
| 29 | Lutete et al., 2021 (29) | Cohort event-monitoring | 5 African countries | 8542 patients | — | — | Pyronaridine–artesunate | PCR day-28 98.6% per-protocol | ROBINS-I |
| 30 | 4ABC Study Group, 2011 (30) | Pediatric RCT | 5 African countries | 4116 children | — | — | AL/ASAQ/DHA-PPQ | Day-28 95.5% / 96.8% / 97.3% | Low (RoB 2.0) |
| 31 | Koko et al., 2025 (31) | Efficacy surveillance | Liberia 2022-2023 | 469 patients | — | — | AL/ASAQ | Day-28 PCR 100%/95.9% and 100%/94.4% | ROBINS-I |
| 32 | Watson et al., 2024 (32) | Risk modelling | Sub-Saharan Africa | — | — | — | — | ACT failure ≈ 31% by 2060 | High |

## Annex D Risk-of-Bias Assessment (RoB 2.0 / Newcastle–Ottawa / RITAM)

### Section D.1 RoB 2.0 Domains (randomised trials)

| Domain | Description | Audit signal-questions |
| --- | --- | --- |
| D1 | Randomisation process | Sequence generation; allocation concealment |
| D2 | Deviations from intended interventions | Participant/personnel blinding; outcome assessor blinding |
| D3 | Missing outcome data | Imputation; sensitivity |
| D4 | Outcome measurement | Appropriate method; variability |
| D5 | Selection of the reported result | Protocol accessible; pre-specified results |

### Section D.2 Global Judgement for 21 Records

| # | Study | D1 | D2 | D3 | D4 | D5 | Global judgement |
| --- | --- | --- | --- | --- | --- | --- | --- |
| 2 | Mueller 2004 (9) | Low | Low | Low | Low | Low | **Low (RoB 1.0)** |
| 3 | Blanke 2008 (10) | Low | Low | High (n=19) | Low | Low | **Low (RoB 1.0)** |
| 4 | Magalhães 2016 (11) | Low | High (unblinded) | High (n=7 arm) | Moderate | Low | **Some concerns** |
| 5 | Ogwang 2012 (12) | Moderate (clusters) | Moderate | Low | Low | Low | **Moderate** |
| 6 | Daddy 2017 (13) | High | High | High | High | High | **High** |
| 7 | Suberu 2013 (14) | N/A | N/A | Low | Low | Low | **Excellent** |
| 9 | Elfawal 2012 (16) | High | N/A | Low | Low | Low | **High** |
| 10 | Elfawal 2015 (17) | High | N/A | Low | Low | Low | **High** |
| 12 | Heide 2006 (19) | N/A | N/A | N/A | Low | Low | **Excellent (analytical)** |
| 13 | Weathers & Towler 2012 (20) | N/A | N/A | N/A | Low | Low | **Excellent (analytical)** |
| 16 | Aubouy 2025 (23) | N/A | N/A | N/A | Low | Low | **Excellent (metabolomics)** |
| 20 | Schreidah 2024 (1) | Low | Low | Low | Low | Low | **High regional** |
| 21 | Uwimana 2020 (2) | Low | Low | Low | Low | Low | **High** |
| 22 | Ishengoma 2024 (4) | Low | Low | Low | Low | Low | **High** |
| 23 | Tumwebaze 2022 (5) | Low | Low | Low | Low | Low | **High** |
| 24 | Katairo 2026 (3) | Low | Low | Low | Low | Low | **High** |
| 25 | Meier-Scherling 2024 (6) | Low | N/A | Low | Low | Low | **High (modelling)** |
| 26 | Schmedes 2021 (27) | Low | Low | Low | Low | Low | **High** |
| 27 | van Loon 2025 (28) | Low | Low | Low | Low | Low | **High** |
| 30 | 4ABC Study 2011 (30) | Low | Low | Low | Low | Low | **Low (RoB 2.0)** |
| 31 | Koko 2025 (31) | Low | Low | Low | Low | Low | **ROBINS-I low/moderate** |

### Section D.3 RITAM Application

RITAM score adds three phytomedicine-specific dimensions not covered by RoB / NOS:

1. **Cultivar variability** (artemisinin per litre): coded High / Medium / Low
2. **Adulteration risk**: systematically reported
3. **Preparation heterogeneity** (leaf/water ratio, decoction time, post-harvest drying conditions): coded High / Medium / Low

## Annex E Exclusions Ledger (46 at screening + 10 at eligibility)

### Section E.1 Exclusions at Screening (n=46, grouped by reason)

| Exclusion category | n | Justification |
| --- | --- | --- |
| Pure artemisinin derivatives without whole-plant comparator | 14 | Outside PICOS C (comparator absent) |
| Non-malarial infection | 8 | Outside PICOS P (population) |
| Artemisia species other than A. annua | 7 | Outside PICOS I (intervention) |
| Veterinary / agricultural studies | 9 | Outside PICOS S (design) |
| Non-empirical literature (commentaries, editorials, isolated abstracts) | 8 | Outside PICOS S (design) |
| **Total** | **46** | — |

### Section E.2 Exclusions at Full-Text Eligibility (n=10, E1–E10 individuals)

| # | Reference | Exclusion reason |
| --- | --- | --- |
| E1 | Study A — pure derivatives | Isolated artemisinin focus without whole-plant A. annua comparator |
| E2 | Study B — pure derivatives | Same |
| E3 | Study C — pure derivatives | Same |
| E4 | Study D — pure derivatives | Same |
| E5 | Study E — non-standardised preparation | Infusion protocol insufficiently reported for replication |
| E6 | Study F — non-standardised preparation | Same |
| E7 | Study G — insufficient follow-up | No day-28 data |
| E8 | Study H — insufficient follow-up | Same |
| E9 | Study I — insufficient follow-up | Same |
| E10 | Study J — non-empirical | Narrative commentary without primary data |

## Annex F GRADE Narrative Synthesis (12 Outcomes)

Column headers: # | Outcome | n studies | n patients/models | Risk of bias | Inconsistency | Indirectness | Imprecision | Publication bias | GRADE certainty | Narrative conclusion

| # | Outcome | n studies | n | RoB | Inconsistency | Indirectness | Imprecision | PB | GRADE |
| --- | --- | --- | --- | --- | --- | --- | --- | --- | --- |
| 1 | Parasitaemia clearance day-7 by A. annua infusion | 3 RCTs | 286 (9–11) | Low | Moderate | Low | Moderate | Low | **Moderate** |
| 2 | Parasitaemia clearance day-28 A. annua monotherapy | 2 direct RCTs + 1 extrapolation | 26 direct | Low | High (decoction heterogeneity) | High | High | Low | **Low** |
| 3 | Day-28 clearance ACTs (AL/ASAQ/DHA-PPQ/PA) in Africa | 3 studies | ~13 000 (29–31) | Low | Low | Low | Low | Low | **High** |
| 4 | Risk reduction of multiple episodes under weekly infusion | 1 cluster RCT | 132 (12) | Moderate | N/A | Low | Moderate | Low | **Moderate** |
| 5 | In-vitro synergy (CI<1) artemisinin + flavonoids against Pf3D7 | 2 studies | ≥ 9 molar ratios (14,15) | Low | Low | Moderate (no human in-vivo) | Low | Low | **High** |
| 6 | Resistance emergence dynamics whole-plant vs DHA in Pfkelch13 murine | 1 study | 3 arms (17) | High (murine model) | Low | High (pre-clinical) | Low | Low | **Moderate** |
| 7 | Reduced heme affinity of Pfkelch13-propeller mutants | 1 mechanistic study | n/a (26) | Low | Low | Low | Low | Low | **High** |
| 8 | Geographic expansion of partial Pfkelch13 markers | 6 clusters | > 2000 isolates (1–4,27,28) | Low | Moderate (regional variations) | Low | Low | Low | **High** |
| 9 | Recovery and stability of casticin/artemetin in infusions | 1 analytical study | n/a (20) | Low | Low | Low | Low | Low | **High** |
| 10 | Cultivar variability of artemisinin in commercial preparations | 2 studies | 22 cultivars/sources (19,23) | Low | High | Low | Low | Low | **Moderate** |
| 11 | Overall safety of A. annua (adult population) | 4 RCTs + 1 cluster | 358 (9–12) | Low | Low | Low | Low | Low | **Moderate** |
| 12 | Salvage efficacy in ACT-refractory severe malaria | 1 case series | 18 (13) | High (cases) | N/A | High | High | High | **Very low** |

## Annex G PROSPERO Registration Metadata

| PROSPERO Field | Reported value |
| --- | --- |
| Register | International Prospective Register of Systematic Reviews, Centre for Reviews and Dissemination, University of York |
| Registration number | **CRD420251128231** |
| Registration date | 25 November 2025 |
| Last-update date | 15 December 2025 |
| Original protocol language | English |
| Public URL | https://www.crd.york.ac.uk/PROSPERO/view/CRD420251128231 |
| Review title | Phytochemical Synergy in Artemisia annua Herbal Tea against Malaria: A Systematic Review of its Efficacy and Safety in the Context of Emerging Pfkelch13 Resistance |
| Review question | Is whole-plant A. annua herbal tea clinically effective and safe in uncomplicated P. falciparum malaria, and does its phytochemical synergy positively modulate the emerging resistance profile under Pfkelch13? |
| Search scope | January 2000 – December 2025; six databases; citation-tracking |
| Population | Patients ≥ 6 months, uncomplicated malaria; pre-clinical models (P. falciparum 3D7 / Dd2 / W2; P. berghei ANKA) |
| Intervention | Whole-plant A. annua infusion, decoction, powdered dried leaves |
| Comparators | ACTs (AL/ASAQ/DHA-PQ/ASMQ/PA), quinine, SP, chloroquine, purified artemisinin |
| Outcomes | Clearance D3/D7/D14/D28; recrudescence; fever; IC₅₀; CI (Chou–Talalay); pharmacokinetics; Pfkelch13 markers (A675V, R561H, C469Y, etc.) |
| Study designs | RCTs, quasi-experimental, prospective/retrospective cohorts, in-vitro studies, murine models |
| Quality-assessment instruments | RoB 2.0; Newcastle–Ottawa; RITAM; narrative GRADE |
| Conflicts of interest | None declared |
| Synthesis strategy | Qualitative narrative synthesis (no pooled meta-analysis) |

## Annex H Controlled Vocabulary, MeSH/EMA Nomenclature, Expanded Abbreviations

### Section H.1 MeSH/EMA Mapping for Key Terms

| Manuscript term | MeSH ID | EMA preferred term | PRISMA mapping |
| --- | --- | --- | --- |
| Artemisia annua | D031150 | Artemisia annua L. herba | Intervention C1 |
| Pfkelch13 | D000071304 + D012320 | Plasmodium falciparum kelch-13 propeller | Outcome 8 |
| Combination Index (Chou–Talalay) | D004305 | Combination index | Outcome 5 |
| Reactive oxygen species | D017382 | ROS / oxidative stress | Mechanism §3.3 |
| Phytochemical matrix | D051548 | Herbal substance; matrix effect | §3.5 |
| SERCA-type Ca²⁺-ATPase | D027701 + D018088 | SERCA Ca²⁺-ATPase | §3.3 (alkylation) |
| Histidine-Rich Protein 2/3 deletion | D064113 | HRP2/HRP3 gene deletion | §3.4 |
| PRISMA 2020 | D000072716 | Reporting guideline | Annex A |

### Section H.2 Expanded Nomenclature for Manuscript Abbreviations

| # | Abbreviation | Full form | MeSH/EMA form |
| --- | --- | --- | --- |
| 1 | ACT | artemisinin-based combination therapy | Therapeutic combination |
| 11 | ConPhyMP | Consortium for Phytotherapeutic Manufacturing Practices | Non-MeSH; WHO-led |
| 23 | h | hour(s) | — |
| 27 | HRP-2 | histidine-rich protein-2 | HRP2 antigen |
| 28 | HRP-3 | histidine-rich protein-3 | HRP3 antigen |
| 29 | IC₅₀ | half-maximal inhibitory concentration | Inhibitory concentration 50 |
| 32 | kg / L / ml | kilogram(s) / litre(s) / millilitre(s) | SI units |
| 60 | PfATP6 | P. falciparum sarcoplasmic/endoplasmic reticulum Ca²⁺-ATPase | SERCA PfATP6 |
| 62 | Pfkelch13 | P. falciparum Kelch-13 propeller | K13 mutation |
| 63 | Pfmdr1 | P. falciparum multidrug-resistance transporter 1 | MDR1 transporter |
| 73 | RDT | rapid diagnostic test | Diagnostic test, point-of-care |
| 74 | RITAM | Research Initiative on Traditional Antimalarial Methods | — |
| 81 | SFE-CO₂ | supercritical fluid extraction with carbon dioxide | Extraction method |
| 86 | UNICAMP CPQBA | Universidade Estadual de Campinas, Centro Pluridisciplinar de Pesquisas Químicas, Biológicas e Agrícolas | Institution |
| 90 | WHO | World Health Organization | — |

### Section H.3 MeSH/PRISMA Reproducibility Checklist for Query Replication

All MeSH descriptors used in Annex B are reproduced here so that any external examiner can reproduce the Boolean chains exactly:

- Artemisia [MeSH]
- Plant Preparations [MeSH]
- Phytotherapy [MeSH]
- Malaria [MeSH]
- Plasmodium falciparum [MeSH]
- Drug Synergism [MeSH]
- Drug Resistance [MeSH]
- Treatment Outcome [MeSH]
- Safety [MeSH]
